# Synthesis and crystal structures of two related Co and Mn complexes: a celebration of collaboration between the universities of Dakar and Southampton

**DOI:** 10.1107/S2056989023009805

**Published:** 2023-11-16

**Authors:** James B. Orton, Ngone Diouf, Rokhaya S. Gueye, Mohamed Gaye, Ibrahima Elhadji Thiam, Simon J. Coles

**Affiliations:** a National Crystallography Service (NCS), Department of Chemistry, University of Southampton, SO17 1BJ, United Kingdom; b Laboratoire de Chimie de Coordination Organique (LCCO), Department of Chemistry, University of Dakar, PB 5005 Dakar, Senegal; Universidade de Sâo Paulo, Brazil

**Keywords:** Africa–UK collaboration, AfCA–IUCr Virtual Collection, manganese complex, cobalt complex, structural chemistry, crystal structure

## Abstract

We report the synthesis and structures of two transition-metal complexes involving 2-(2-hy­droxy­phen­yl)benzimidazole (2hpbi – a ligand of inter­est for its photoluminescent applications), with cobalt, namely, bis­[μ-2-(1*H*-1,3-benzo­diazol-2-yl)phenolato]bis­[ethanol(thio­cyanato)­cobalt(II)], [Co_2_(C_13_H_9_N_2_O)_2_(NCS)_2_(C_2_H_6_O)_2_], (**1**), and manganese, namely, bis­[μ-2-(1*H*-1,3-benzo­diazol-2-yl)phenolato]bis­{[2-(1*H*-1,3-benzo­diazol-2-yl)phenolato](thio­cyanato)­mang­an­ese(III)} dihydrate, [Mn_2_(C_13_H_9_N_2_O)_4_(NCS)_2_]·2H_2_O, (**2**). These structures are two recent examples of a fruitful collaboration between researchers at the Laboratoire de Chimie de Coordination Organique/Organic Coordination Chemistry Laboratory (LCCO), University of Dakar, Senegal and the National Crystallography Service (NCS), School of Chemistry, University Southampton, UK. This productive partnership was forged through meeting at Pan-African Conferences on Crystallography and quickly grew as the plans for the AfCA (African Crystallographic Association) developed. This article therefore also showcases this productive partnership, in celebration of the IUCr’s 75 year anniversary and the recent inclusion of AfCA as a Regional Associate of the IUCr.

## Abstract 2

The synthesis and structures are reported of two transition-metal complexes involving 2-(2-hy­droxy­phen­yl)benzimidazole with cobalt and manganese, arising from a UK–Africa collaboration.

## 1. Chemical context

One of the conclusions from the Pan African Summit meeting (15–17^th^ October 2014, University of the Free State, Bloemfontein, South Africa; Roodt *et al.*, 2014 ▸) held during the Inter­national Year of Crystallography, IYCr2014, was the lack of convenient crystallographic facilities across the continent. It was also recognised that crystallography is not well developed or studied in most of Africa and that opportunities for crystallographic researchers to inter­act with each other, as well as to those outside of the continent, are sparse. As a result, teaching activities are quite rare and the chance to develop new skills, competencies and capabilities, remained low.

To foster the progress of both practical and theoretical expertise within and around crystallography, programmes such as the IUCr Crystallography in Africa Initiative (Lecomte *et al.*, 2014 ▸) aim to promote and develop crystallography (both teaching and research), throughout the continent. A primary goal is to create and support the operation of at least one national crystallography centre per country, ultimately to enable each of these to become independent hub of research in fields related to crystallography and the structural sciences. As part of the Inter­national Year of Crystallography activities, the IUCr–UNESCO OpenLab network was launched. This was a global activity to provide a network of functioning laboratories across developing nations, thereby enabling access to crystallographic instrumentation to a much broader range of researchers. In Africa, OpenLab was operated within the framework of the IUCr Crystallography in Africa initiative and acted as a starting point for a much wider range of researchers to fully engage with crystallographic experimentation.

Establishing the first Pan-African Crystallography Conference (PCCr1: Dschang, Cameroon, 2016) was a notable milestone towards the goal of coalescing crystallography-based research in Africa. In fact, its successes went much further than this, placing African crystallography on the world stage and attracting a notable number of delegates from further afield. Of particular success was the promotion of networking between delegates, both from within Africa and to those attending from all around the world. Subsequently PCCr2 and PCCr3 have been held in Accra, Ghana (2019) and Nairobi, Kenya (2023), respectively, have built on the platform established by PCCr1, resulting in far greater mobility, collaboration and empowerment for African crystallographers.

The Laboratoire de Chimie de Coordination Organique (LCCO) is a research unit linked to the Department of Chemistry at the University of Dakar, Senegal. It opened in 1995, under the guidance of Prof. Abdou Salam Sall (the former Rector of the University of Dakar) and Prof. Mohamed Lamine Gaye with a focus on structural chemistry, inorganic chemical synthesis, anti­oxidant activity, X-ray diffraction and spectroscopic characterization. Since its inception, over 20 students have successfully completed their PhD programs. It also continues to ramp up its research capacity, by expanding its complement of staff, both locally and with some based at other universities within Senegal. This expansion reflects the wider University strategy, which has seen the University of Dakar being declared the best university in Francophone Sub-Saharan Africa and the top-rated higher education institution in Senegal (by several independent online metric-based ranking sites, such as edurank.org, webmetrics.info *etc*.).

Over its 40+ year history (Hursthouse *et al.*, 2014 ▸), the National Crystallography Service (NCS) has provided access, primarily for UK researchers, to advanced crystallographic facilities and expertise to support those lacking facilities or to those with very challenging samples. With a strong history championing and investing into diffraction technology innovations, the NCS supports users from a wide variety of disciplines from over 30 different academic institutions in addition to several commercial clients. It also holds several teaching and outreach events each year, to promote science in general, and crystallography in particular. It is therefore well-placed to contribute (in part) to the development of African crystallography, by making some of these opportunities available. NCS has therefore been very fortunate to be involved in some of the significant development activities and has relished the opportunities that have since arisen, to collaborate closely with a range of new colleagues across Africa.

Although a connection had already been made, a strong collaboration between the LCCO and the NCS grew out of meeting face-to-face at PCCr2. Later in 2019, Dr Thiam undertook a two-week secondment to the NCS in Southampton, which cemented the basis of the collaboration. This visit was a valuable training opportunity covering crystallographic instrumentation and software, sample selection and handling, screening, collection strategy, data processing, structure solution and publication preparation. During the visit, over 20 full datasets were collected. This activity enabled considerable knowledge transfer to LCCO and subsequently, more than 70 further sample analyses have been successfully completed. Many novel and inter­esting structures have been characterized and have resulted in eleven joint publications in a range of journals, between 2018 and 2023 (Gaye *et al.*, 2018, 2020 ▸; Gaye, Fall *et al.*, 2023 ▸; Gaye, Sarr *et al.*, 2023 ▸; Gaye, Ndoye *et al.*, 2021 ▸; Gaye, Kebe *et al.*, 2021 ▸; Sokhna *et al.*, 2023 ▸); Diallo *et al.*, 2022 ▸; Diop *et al.*, 2019 ▸; Faye *et al.*, 2020 ▸; Sarr *et al.*, 2018 ▸; Sylla-Gueye *et al.*, 2020 ▸). Recent samples originated from more than a dozen current doctoral students from within the LCCO itself, the wider Department of Chemistry or from two additional joint projects with the University of Bambey, Senegal and Gaston Berger University of Saint-Louis, Senegal.

The volume of crystal structures being produced *via* this collaboration is significant, particularly for an African-based group. This means it is not possible, or necessary, to publish all these in the primary literature. Accordingly, we have made over 20 CSD Communications (see https://www.ccdc.cam.ac.uk/community/access-deposit-structures/deposit-a-structure/csd-communications/), as part of an ongoing exercise.

2-(2-Hy­droxy­phen­yl)benzimidazoles are of inter­est for their photoluminescence properties and how the optical properties are effected by chelation to various metal centres has been previously reported (Zheng *et al.*, 2003 ▸; Tong *et al.*, 2005*a* ▸,*b* ▸). Exploration of these systems, particularly how the choice of metal centre and how modification of the coordination sphere may effect both crystal packing and the photoluminescence response, is of ongoing inter­est. Herein we report two recent structures resulting from this study. 
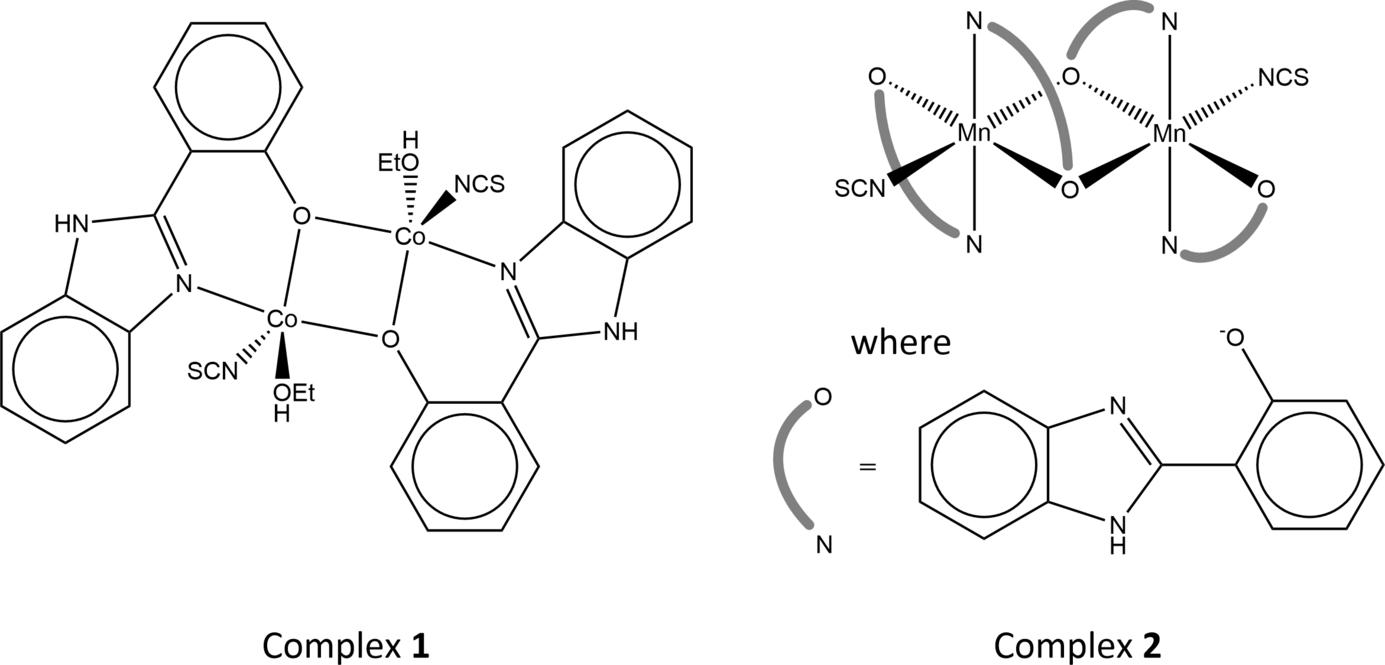


## 2. Structural commentary

**Figure 1:**
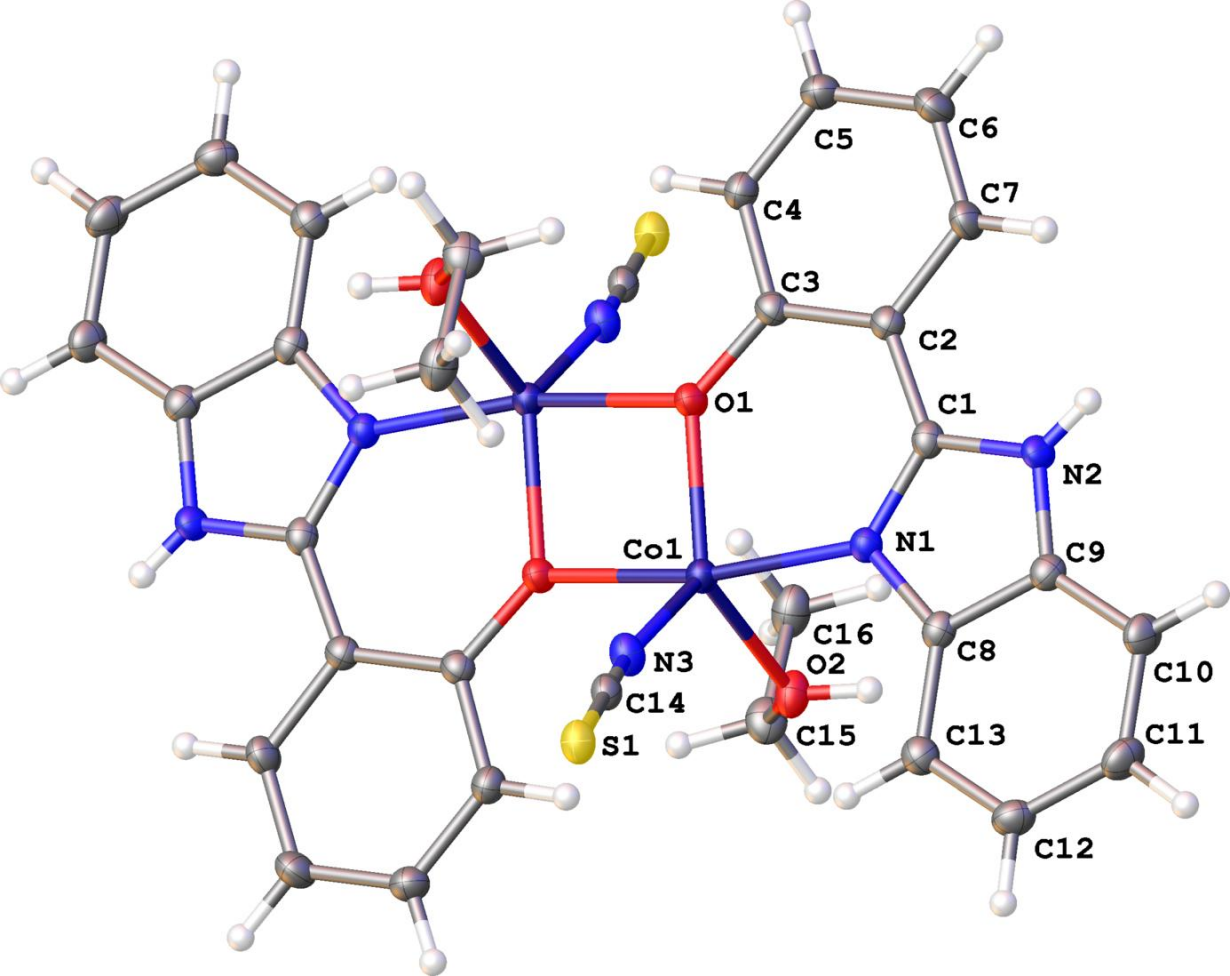
Bi-nuclear Co^II^ complex of **1**, ellipsoids shown at 50% probability.

Complex **1** is a *Z* = 0.5 structure in *P*2_1_/*c*, comprised of a discrete μ_2_ oxo-bridged bi-nuclear complex of Co^II^ ions [Co⋯Co = 3.1326 (6) Å], with each metal centre being five-coordinate (trigonal bipyramidal), with a trigonality (or Addison) index of τ = 0.82 (Addison *et al.*, 1984 ▸) denoting a slight deformation away from an idealized geometry (further details given in the supporting information). Their coordination spheres (related through an inversion centre), include one SCN^−^, one EtOH and a 2hpbi ligand, through its N1 and O1 (bridging) atoms, as shown in Fig. 1 ▸.

**Figure 2:**
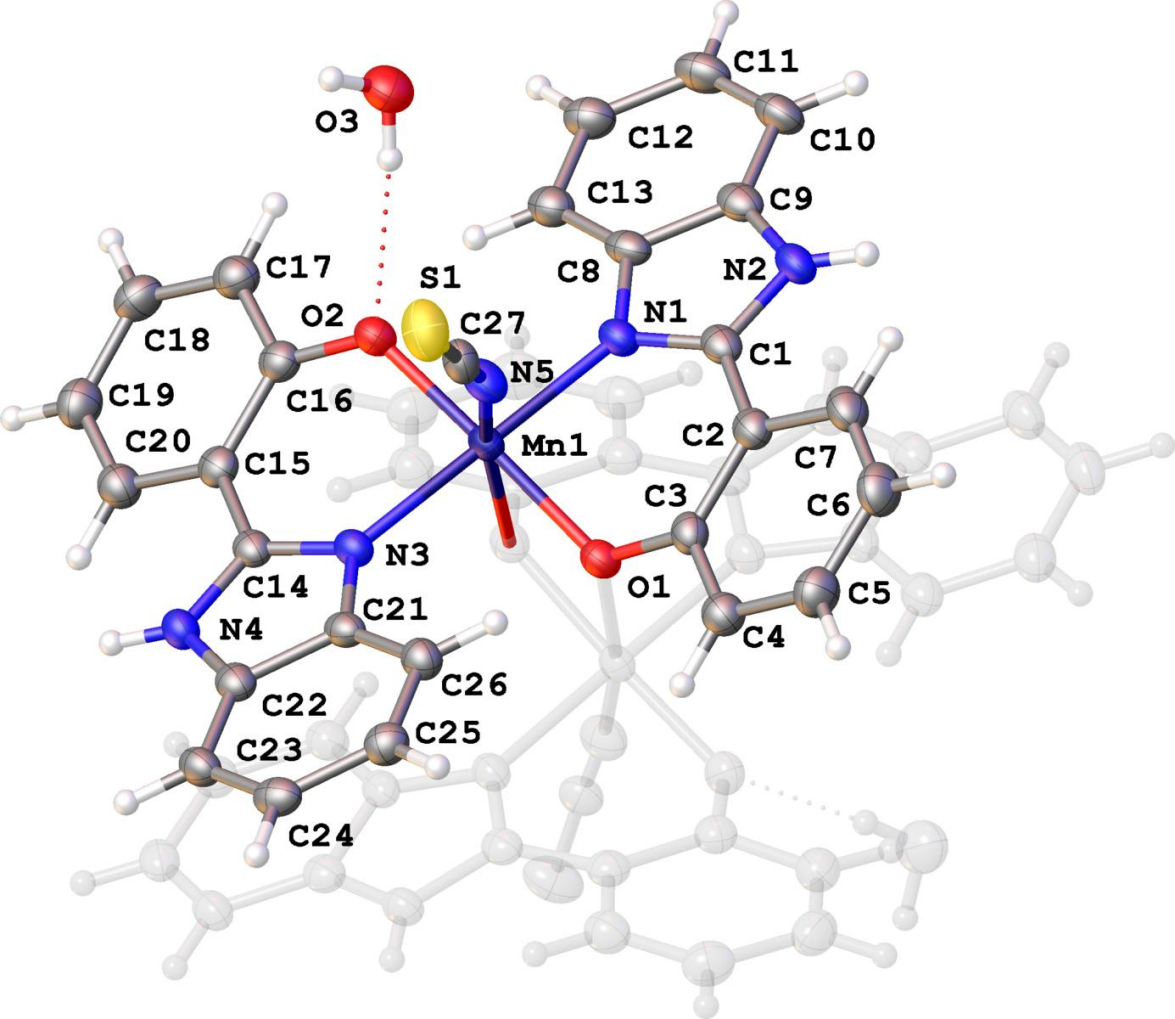
Bi-nuclear Mn^III^ complex of **2**, ellipsoids shown at 50% probability and with symmetry-equivalent atoms ghosted.

Similarly, **2** is also a *Z* = 0.5 structure, although in this case in the space group *C*2/*c*. The complex also comprises a discrete μ_2_ oxo-bridged bi-nuclear complex; however, the metal centres have undergone oxidation (during formation, in air), from Mn^II^ to Mn^III^ [Mn⋯Mn = 3.3769 (7) Å]. Both Mn ions are six-coordinate (octa­hedral) and their coordination spheres are related by rotational symmetry. Each Mn^III^ coordinates to one SCN^−^ and two 2hpbi ligands, one *via* atoms N1 and O1 (bridging) and the other through atoms N3 and O2 (Fig. 2 ▸).

## 3. Supra­molecular features

**Figure 3:**
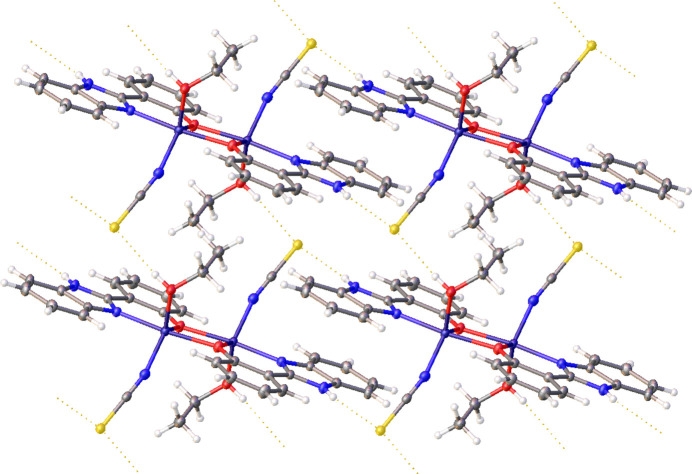
Hydrogen-bonding network within **1**, ellipsoids shown at 50% probability.

Packing in **1** is directed by a 2-D network of N—H⋯S and O—H⋯S hydrogen bonds (Table 1 ▸), depicted in Fig. 3 ▸. Comparison with similar examples (CSD-Materials – CSD v5.44, Apr 2023; Macrae *et al.*, 2020 ▸) confirmed that these inter­actions were typical of their type (summarized in S1; for further details, see supporting information section SI-1).

**Figure 4:**
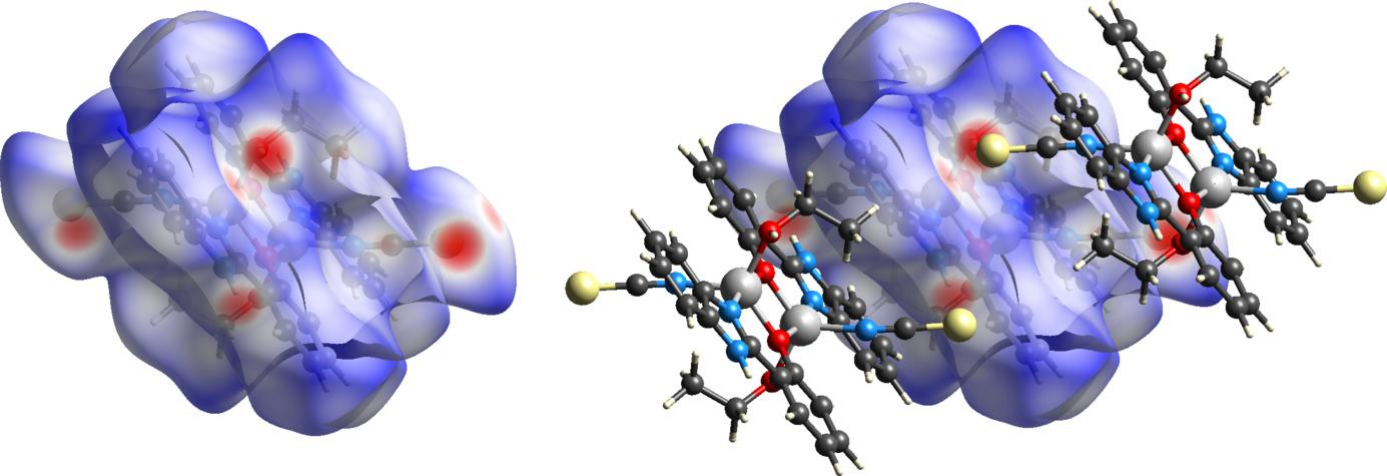
Hirshfeld surfaces for **1** mapped over *d*_norm_ in the range −0.44 Å (red) to 1.41 Å (blue).

**Figure 5:**
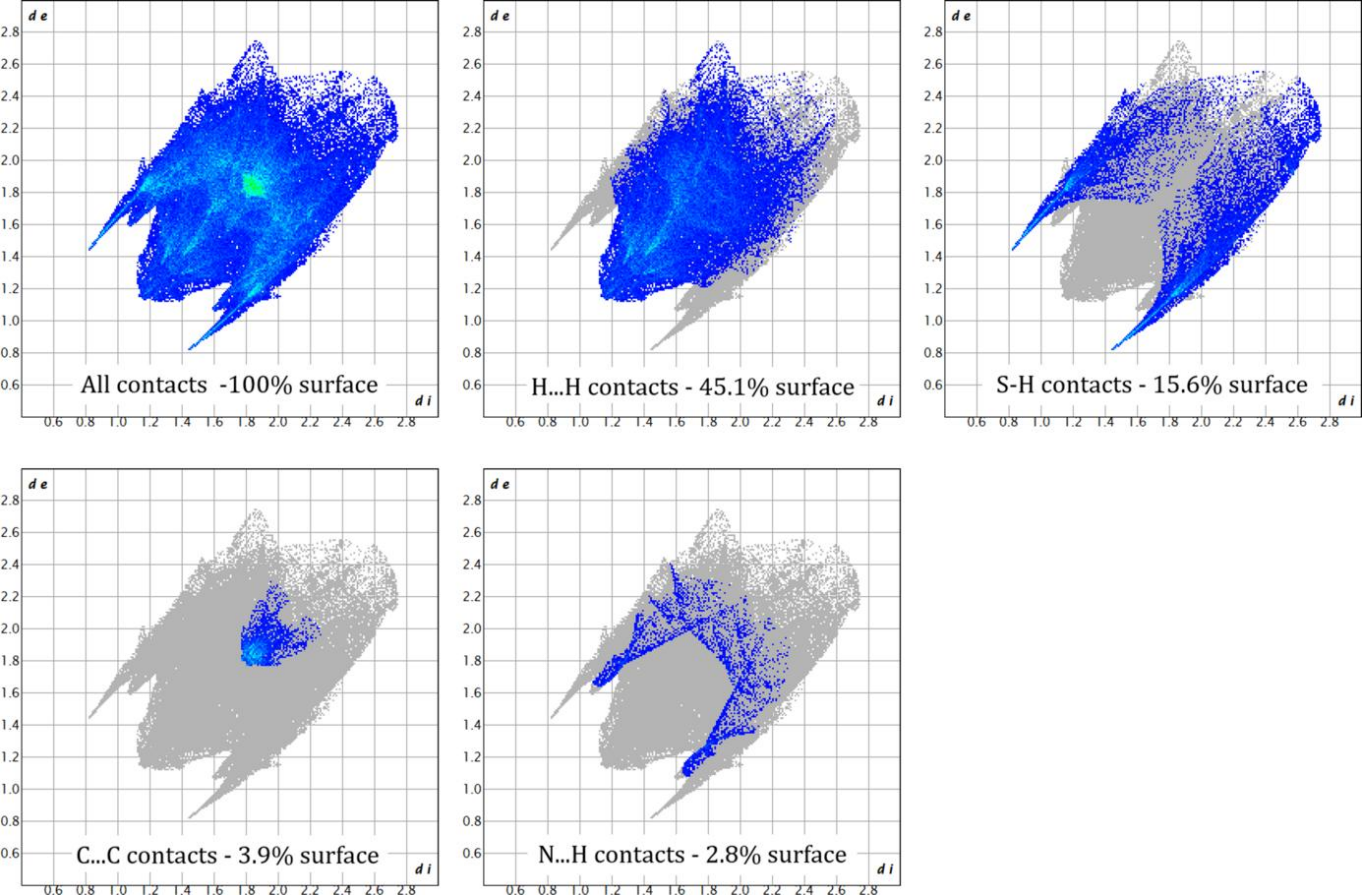
Two-dimensional fingerprint plots of **1**.

Analysis of the Hirshfeld surface and associated fingerprint plots (Spackman *et al.*, 2021 ▸) of complex **1** provide further evidence for these contacts. Fig. 4 ▸ shows the Hirshfeld surface mapped over *d*_norm_ (normalized contact distance) in the range −0.4413 Å (red) to 1.4142 Å (blue), where donors and acceptors of these contacts are the bright-red areas. Two-dimensional fingerprint plots are shown in Fig. 5 ▸, with characteristic sharp features arising from hydrogen bonding being present.

**Figure 6:**
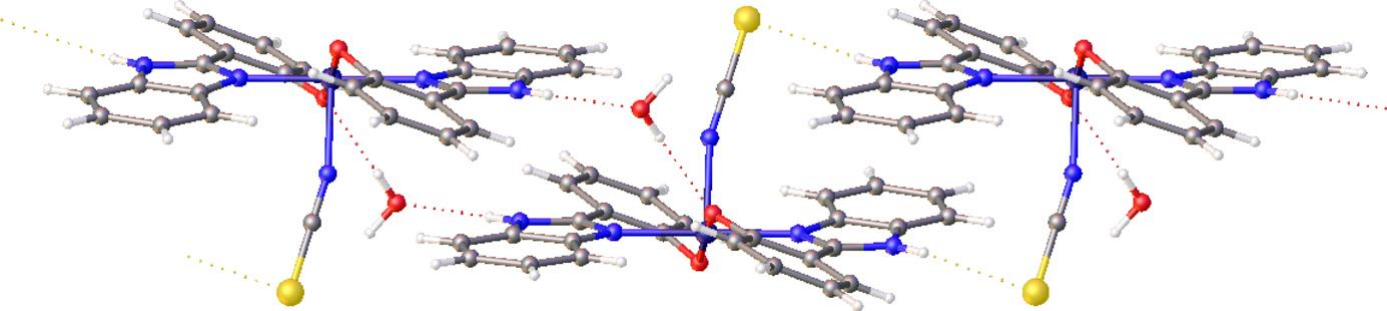
Hydrogen bonding network within **2**, ellipsoids shown at 50% probability.

The contacts directing the crystal packing in **2** identified a more complex mixture of hydrogen bonds: O—H⋯O and O—H⋯N involving the included solvent water and an N—H⋯S contact, of type similar to that seen in **1**. These collectively form a 1D chain-like array, Fig. 6 ▸, summarized in Table 2 ▸ and with further details in supporting information section SI-2.

**Figure 7:**
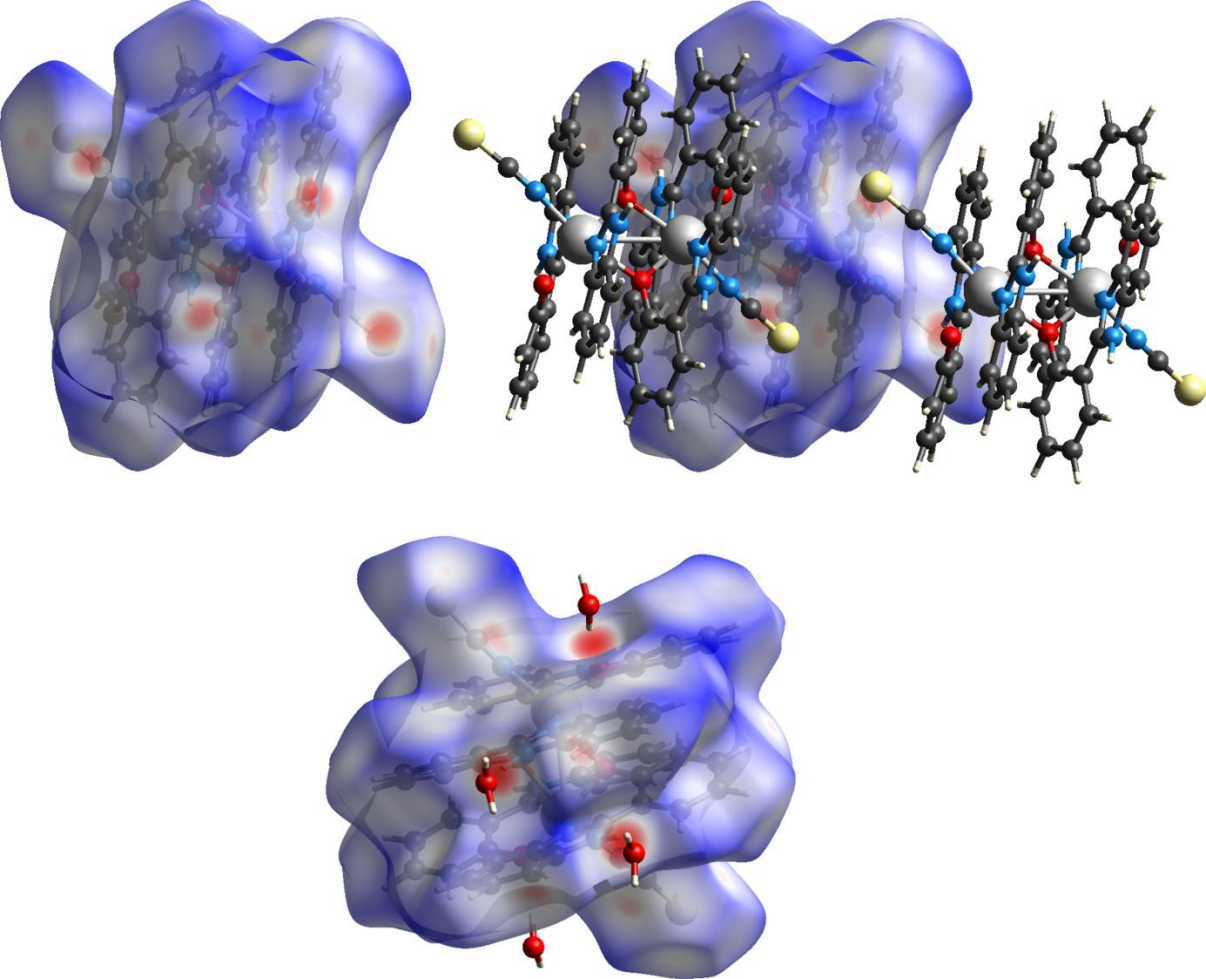
Hirshfeld surfaces for **2** mapped over *d*_norm_ in the range −0.49 Å (red) to 1.56 Å (blue).

**Figure 8:**
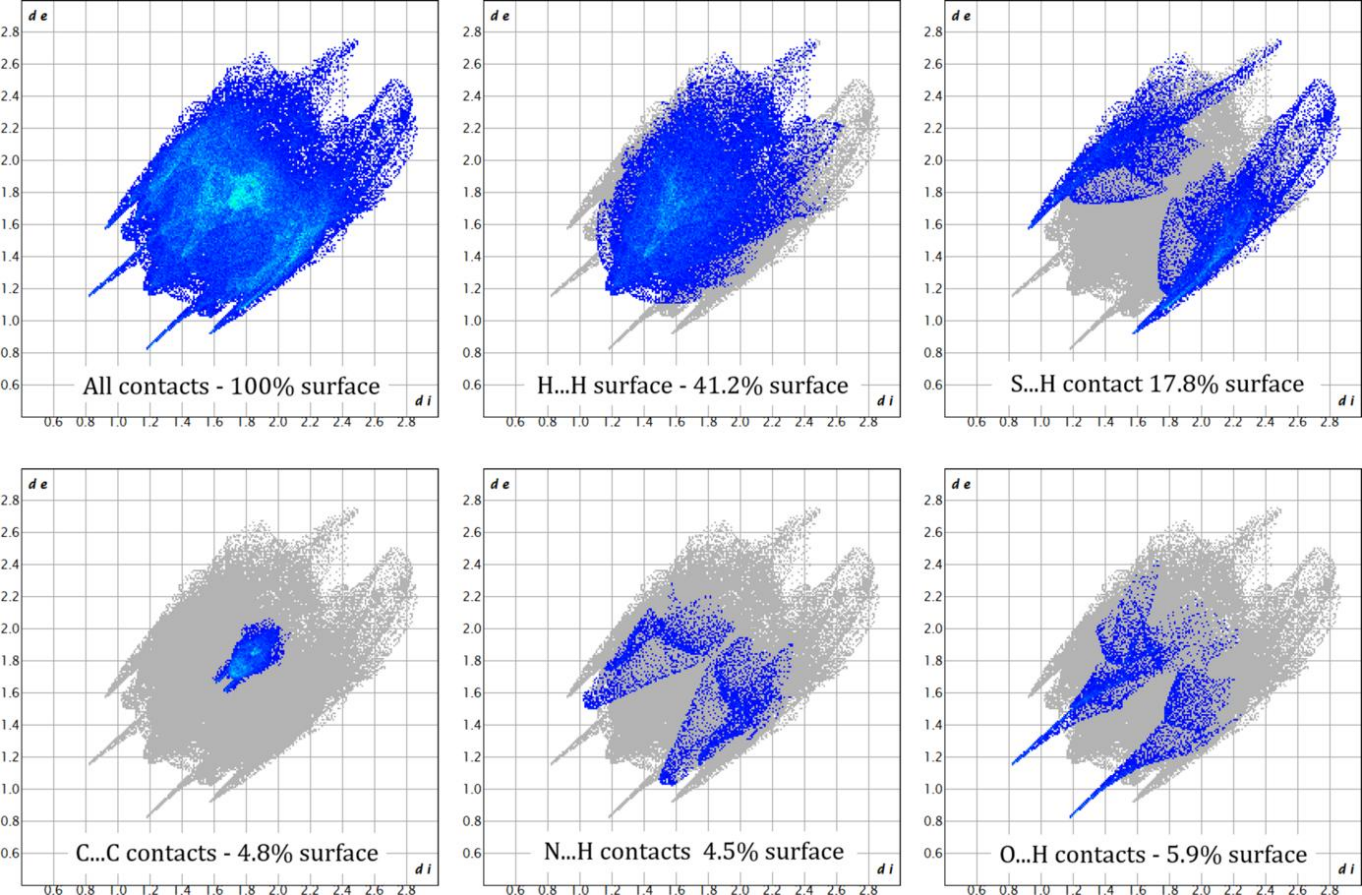
Two-dimensional fingerprint plots of **2.**

Fig. 7 ▸ shows the Hirshfeld surface of complex **2**, mapped over *d*_norm_ range −0.4884 Å (red) to 1.5602 Å (blue), again highlighting the hydrogen bonding present. Two-dimensional fingerprint plots are shown in Fig. 8 ▸, which, when compared to similar contacts in the CSD, provide further insight into the crystal packing. The strongest and sharpest features corres­pond to the O—H⋯O(water) contacts, which are now both the strongest hydrogen-bond donor and acceptor in the structure. The N—H⋯S features are noticeably weaker than in structure **1**, reflected in both its relative lengthening (0.3 Å) and being further perturbed from a linear geometry (163 to 152°).

Also of minor note, the second hydrogen (H3*B*) of the solvent water (containing O3) is orientated towards atom S1 of an adjacent complex as though to form a hydrogen bond. The distance, however (3.8 Å), is much longer than the bulk of comparable contacts in the CSD (3.2–3.4 Å) and its position is simply optimized for steric packing.

## 4. Database survey

A CSD search (v5.44, Apr 2023; Groom *et al.*, 2016 ▸) for complexes involving the (unfunctionalized) 2hpbi ligand returned 69 hits, nine of which are coordinated by Co or Mn and none containing thio­cyanates. Eight of these Co or Mn complex species consist of a single metal centre, with the remaining structure, PULGUZ (Duan *et al.*, 2010 ▸), forming a bi-nuclear complex, though quite dissimilar in structure to **2**. PULGUZ contains two six-coordinate Mn^IV^ centres, each chelated by two 2hpbi ligands then bridged by two O^2−^ ions [Mn⋯Mn = 2.7772 (9) Å].

## 5. Synthesis and crystallisation

The 2hpbi ligand, [2-(1*H*-benzimidazol-2-yl)phenol (C_13_H_10_N_2_O)], prepared by the slow addition (∼2 h, *via* dropping funnel) of 2-hy­droxy­benzaldehyde (30mmol, in ethanol) into a flask containing ortho­phenyldi­amine (30mmol, in ethanol), in the presence of few drops of glacial acetic acid. On cooling, no precipitate was observed and the reaction mixture was evaporated to dryness, to give a yellow oil. A yellow precipitate was obtained on the addition of diethyl ether, which was separated and thoroughly washed with further ether, then dried over P_4_O_10_. FT–IR and NMR spectra for the 2hpbi ligand are consistent to those reported (values below, further details in supporting information section SI-3), yield 93%.

FT–IR (*ν*, cm-1): (C=N) 1605, (C—O) 1278, (OH) 3431, (NH) 3344, (C=C)_ar_ 1528, 1460, 1489. ^1^H NMR (300 MHz, δ (ppm), Acetone-*d*_6_): 7.85 (*s*, 1H, –NH), 7.82 (*s*, 1H, –OH), 6,91–7.51 (*m*, 8H, H_ar_). ^13^C NMR (75 MHz, δ (ppm), Acetone-*d*_6_): 205.93 (C-11), 159.79 (C-8), 152.88 (C-4 & C-5), 132.56 (C-13 & C-15), 126.52 (C-1 & C-2), 123.86 (C-14), 119.82 (C-3 & C-12), 118.28 (C-6), 113.48 (C-10). **^1^** ^3^C NMR DEPT-135 (75 MHz, δ (ppm), Acetone-*d*_6_) disappearance of signals: 205.93 (C-11), 159.79 (C-8), 152.88 (C-4 et C-5), 113.48 (C-10).

Crystallization from ethanol and slow evaporation led to the formation of crystals suitable for single crystal X-ray diffraction, after ∼1 week. Transition-metal complexes **1** and **2** were synthesized by first suspending the respective metal salt: CoCl_2_·2H_2_O (0.042g, 0.25mmol) or MnCl_2_·2H_2_O (0.040g, 0.25mmol) with NH_4_SCN (0.038g, 0.5mmol) in ethanol (and a few drops of water). These mixtures were then filtered and added to ethanol solutions of ligand 2-(1*H*-benzimidazol-2-yl)phenol (0.053g, 0.25mmol), subsequently stirred for 1 h, then filtered and allowed to slowly evaporate (1 week). In both cases, crystals suitable for scXRD were produced, dark-red crystals of the cobalt complex **1** (m.p. = 495 K) and pale-brown crystals of the manganese complex **2** (m.p. > 533 K). Crystallographic analysis identified **1** as the complex [Co_2_(C_13_H_9_N_2_O)_2_(NSC)_2_(CH_3_CH_2_OH)_2_] and **2** as the complex [Mn_2_(C_13_H_9_N_2_O)_4_(NSC)_2_]·2H_2_O.

## 6. Refinement

Details of the crystal data, data collection and structure refinement are summarized in Table 3 ▸.

Diffraction data were collected using a Rigaku 007HF diffractometer with graphite monochromatized Cu *K*_α_ radiation equipped with Varimax confocal mirrors, a UG2 Universal goniometer, a HyPix Arc-100 detector and an Oxford Cryosystems low-temperature device operating at 100 (2) K. Cell parameters, collection strategy, data reduction (corrected for Lorentz and polarization effects), data integration and adsorption corrections were performed using *CrysAlis PRO* v1.171.42.80a (Rigaku OD, 2023 ▸). The structure was solved with the *SHELXT2018/2* (Sheldrick, 2015*a* ▸) solution program using dual methods within the *OLEX2 1.5* suite of programs (Dolomanov *et al.* 2009 ▸). The model was refined with *SHELXL2018/3* (Sheldrick, 2015*b* ▸) using full-matrix least-squares minimization on *F* ^2^.

C-bound H atoms were positioned geometrically (0.95–0.98 Å) and refined as riding with *U*_iso_(H) = 1.2*U*_eq_(C). Other H atoms were refined with *U*_iso_(H) = 1.2*U*_eq_(N) or 1.5*U*_eq_(O). In **2**, the restraint N2—H2 = N4—H4*A* = 0.88±(2) Å was applied.

## 7. Reflections and Future Work

This collaboration between the universities of Dakar and Southampton is just one of the many ways in which the initiatives promoting crystallography and related fields across African nations are helping to raise the profile of African research. The number of new compounds and crystals produced by LCCO is impressive and the value of these outputs are greatly increased *via* access to crystallographic facilities. The benefits for both institutions are clear when considering the number of new crystal structures generated and that the publication rate averages over two articles per year. The NCS benefits from a number of such collaborations, most of which have stemmed from networking at Pan-African conferences and related events and so we encourage researchers from outside the continent to attend these and engage with the wealth of science being conducted. The outputs from projects supported by the collaboration and the secondment to Southampton both contributed strongly to the recent promotion of Dr Thiam to the rank of Professor.

Finally, the NCS is launching a service for single-crystal structure analysis by Electron Diffraction to complement its established state-of-the-art X-ray facilities. This technique is on the verge of becoming more routine and provides many opportunities for new structural chemistry research and the ability to examine nanocrystals not only extends the capabilities of X-rays, but also opens up our technique to areas of chemistry that could not previously benefit. The NCS is currently exploring routes by which it can make these capabilities available to colleagues in Africa.

## Supplementary Material

Crystal structure: contains datablock(s) global, Struct-1. DOI: 10.1107/S2056989023009805/ex2078sup1.cif

Structure factors: contains datablock(s) Struct-1. DOI: 10.1107/S2056989023009805/ex2078Struct-1sup2.hkl

Structure factors: contains datablock(s) Struct-2. DOI: 10.1107/S2056989023009805/ex2078Struct-2sup3.hkl

H-bonding analysis and spectroscopic information. DOI: 10.1107/S2056989023009805/ex2078sup5.pdf

CCDC references: 2306731, 2306730

Additional supporting information:  crystallographic information; 3D view; checkCIF report

## Figures and Tables

**Table 1:** Hydrogen-bond geometry (Å, °) for **1** [Chem scheme1]

*D*—H⋯*A*	*D*—H	H⋯*A*	*D*⋯*A*	*D*—H⋯*A*
N2—H2⋯S1^i^	0.78 (3)	2.63 (3)	3.3843 (17)	162 (2)
O2—H2*A*⋯S1^ii^	0.84 (3)	2.42 (3)	3.2498 (15)	174 (2)

Symmetry codes: (i) 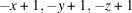 ; (ii) 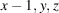 .

**Table 2:** Hydrogen-bond geometry (Å, °) for **2** [Chem scheme1]

*D*—H⋯*A*	*D*—H	H⋯*A*	*D*⋯*A*	*D*—H⋯*A*
N2—H2⋯O3^i^	0.85 (2)	2.11 (2)	2.893 (3)	152 (2)
N4—H4*A*⋯S1^ii^	0.85 (2)	2.64 (2)	3.4147 (19)	152 (2)
O3—H3*A*⋯O2	0.83 (4)	2.17 (4)	2.987 (2)	173 (3)
O3—H3*B*⋯S1^iii^	0.89 (4)	2.94 (4)	3.799 (2)	163 (3)

Symmetry codes: (i) 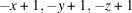 ; (ii) 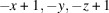 ; (iii) 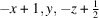 .

**Table 3:** Experimental details

	**1**	**2**
Crystal data		
Chemical formula	[Co_2_(C_13_H_9_N_2_O)_2_(NCS)_2_(C_2_H_6_O)_2_]	[Mn_2_(C_13_H_9_N_2_O)_4_(NCS)_2_]·2H_2_O
*M*_r_	744.60	1098.96
Crystal system, space group	Monoclinic, *P*2_1_/*c*	Monoclinic, *C*2/*c*
Temperature (K)	100	100
*a*, *b*, *c* (Å)	7.74843 (10), 19.2895 (3), 10.69753 (11)	21.8914 (6), 16.3110 (4), 13.5260 (4)
β (°)	93.3777 (10)	100.163 (2)
*V* (Å^3^)	1596.11 (4)	4754.0 (2)
*Z*	2	4
Radiation type	Cu *K*α	Cu *K*α
μ (mm^−1^)	9.76	5.69
Crystal size (mm)	0.12 × 0.1 × 0.1	0.07 × 0.05 × 0.02

Data collection		
Diffractometer	Rigaku 007HF diffractometer with HF Varimax confocal mirrors, an UG2 goniometer and HyPix Arc-100 detector	Rigaku 007HF diffractometer with HF Varimax confocal mirrors, an UG2 goniometer and HyPix Arc-100 detector
Absorption correction	Analytical (*CrysAlis PRO*; Rigaku OD, 2023 ▸)	Gaussian (*CrysAlis PRO*; Rigaku OD, 2023 ▸)
*T*_min_, *T*_max_	0.785, 0.826	0.940, 1.000
No. of measured, independent and observed [*I* > 2σ(*I*)] reflections	46610, 3248, 3113	34537, 4776, 3991
*R*_int_	0.037	0.043

Refinement		
*R*[*F* ^2^ > 2σ(*F* ^2^)], *wR*(*F* ^2^), *S*	0.029, 0.079, 1.08	0.039, 0.107, 1.05
No. of reflections	3248	4776
No. of parameters	215	346
No. of restraints	0	2
H-atom treatment	H atoms treated by a mixture of independent and constrained refinement	H atoms treated by a mixture of independent and constrained refinement
Δρ_max_, Δρ_min_ (e Å^−3^)	0.32, −0.47	1.24, −0.48

Computer programs: *CrysAlis PRO* (Rigaku OD, 2023 ▸), *SHELXT2018/2* (Sheldrick, 2015*a* ▸), *SHELXT2018/2* (Sheldrick, 2015*b* ▸), *SHELXL2018/3* (Sheldrick, 2015*b* ▸), *SHELXL2018/3* (Sheldrick, 2015*a* ▸) and *OLEX2* (Dolomanov *et al.*, 2009 ▸).

## supplementary crystallographic information

### Bis[µ-2-(1H-1,3-benzodiazol-2-yl)phenolato]bis[ethanol(thiocyanato)cobalt(II)] (Struct-1) Crystal data

**Table:**

[Co_2_(C_13_H_9_N_2_O)_2_(NCS)_2_(C_2_H_6_O)_2_]	*F*(000) = 764
*M**_r_* = 744.60	*D*_x_ = 1.549 Mg m^−^^3^
Monoclinic, *P*2_1_/*c*	Cu *K*α radiation, λ = 1.54178 Å
*a* = 7.74843 (10) Å	Cell parameters from 12655 reflections
*b* = 19.2895 (3) Å	θ = 4.6–74.2°
*c* = 10.69753 (11) Å	µ = 9.76 mm^−^^1^
β = 93.3777 (10)°	*T* = 100 K
*V* = 1596.11 (4) Å^3^	(cut) block, dark_red
*Z* = 2	0.12 × 0.1 × 0.1 mm

### Bis[µ-2-(1H-1,3-benzodiazol-2-yl)phenolato]bis[ethanol(thiocyanato)cobalt(II)] (Struct-1) Data collection

**Table:**

Rigaku 007HF diffractometer with HF Varimax confocal mirrors, an UG2 goniometer and HyPix Arc-100 detector	3248 independent reflections
Radiation source: Rotating anode, Rigaku 007 HF	3113 reflections with *I* > 2σ(*I*)
HF Varimax focusing mirrors monochromator	*R*_int_ = 0.037
Detector resolution: 10 pixels mm^-1^	θ_max_ = 74.5°, θ_min_ = 4.6°
profile data from ω–scans	*h* = −9→9
Absorption correction: analytical (CrysAlisPro; Rigaku OD, 2023)	*k* = −24→24
*T*_min_ = 0.785, *T*_max_ = 0.826	*l* = −13→9
46610 measured reflections	

### Bis[µ-2-(1H-1,3-benzodiazol-2-yl)phenolato]bis[ethanol(thiocyanato)cobalt(II)] (Struct-1) Refinement

**Table:**

Refinement on *F*^2^	Primary atom site location: dual
Least-squares matrix: full	Hydrogen site location: mixed
*R*[*F*^2^ > 2σ(*F*^2^)] = 0.029	H atoms treated by a mixture of independent and constrained refinement
*wR*(*F*^2^) = 0.079	*w* = 1/[σ^2^(*F*_o_^2^) + (0.0428*P*)^2^ + 1.1982*P*] where *P* = (*F*_o_^2^ + 2*F*_c_^2^)/3
*S* = 1.08	(Δ/σ)_max_ = 0.001
3248 reflections	Δρ_max_ = 0.32 e Å^−^^3^
215 parameters	Δρ_min_ = −0.47 e Å^−^^3^
0 restraints	

### Bis[µ-2-(1H-1,3-benzodiazol-2-yl)phenolato]bis[ethanol(thiocyanato)cobalt(II)] (Struct-1) Special details

**Table:**

Geometry. All esds (except the esd in the dihedral angle between two l.s. planes) are estimated using the full covariance matrix. The cell esds are taken into account individually in the estimation of esds in distances, angles and torsion angles; correlations between esds in cell parameters are only used when they are defined by crystal symmetry. An approximate (isotropic) treatment of cell esds is used for estimating esds involving l.s. planes.

### Bis[µ-2-(1H-1,3-benzodiazol-2-yl)phenolato]bis[ethanol(thiocyanato)cobalt(II)] (Struct-1) Fractional atomic coordinates and isotropic or equivalent isotropic displacement parameters (Å^2^)

**Table:**

	*x*	*y*	*z*	*U*_iso_*/*U*_eq_	
C1	0.3063 (2)	0.52949 (10)	0.66344 (16)	0.0155 (3)	
C2	0.2850 (2)	0.59244 (9)	0.73665 (16)	0.0162 (3)	
C3	0.3522 (2)	0.59966 (9)	0.86219 (16)	0.0160 (3)	
C4	0.3285 (2)	0.66357 (10)	0.92215 (17)	0.0191 (4)	
H4	0.374718	0.669594	1.005696	0.023*	
C5	0.2404 (3)	0.71778 (10)	0.86349 (18)	0.0214 (4)	
H5	0.225874	0.760134	0.906971	0.026*	
C6	0.1726 (3)	0.71056 (10)	0.74058 (19)	0.0243 (4)	
H6	0.111657	0.747697	0.699798	0.029*	
C7	0.1954 (3)	0.64873 (10)	0.67909 (17)	0.0205 (4)	
H7	0.149354	0.643886	0.595268	0.025*	
C8	0.3772 (2)	0.42614 (10)	0.60066 (16)	0.0160 (3)	
C9	0.2867 (2)	0.45834 (9)	0.49991 (17)	0.0171 (4)	
C10	0.2497 (3)	0.42482 (10)	0.38593 (17)	0.0221 (4)	
H10	0.187768	0.447169	0.318040	0.027*	
C11	0.3079 (3)	0.35727 (11)	0.37699 (18)	0.0240 (4)	
H11	0.284963	0.332479	0.301094	0.029*	
C12	0.4001 (3)	0.32444 (10)	0.47743 (18)	0.0218 (4)	
H12	0.439085	0.278180	0.467481	0.026*	
C13	0.4355 (2)	0.35798 (10)	0.59071 (17)	0.0198 (4)	
H13	0.496974	0.335530	0.658703	0.024*	
C14	0.8273 (2)	0.40481 (9)	0.76185 (17)	0.0172 (4)	
C15	0.2158 (3)	0.35669 (11)	1.00665 (18)	0.0237 (4)	
H15A	0.304462	0.344454	1.073436	0.028*	
H15B	0.145670	0.314781	0.986900	0.028*	
C16	0.1014 (3)	0.41258 (12)	1.05322 (19)	0.0273 (4)	
H16A	0.011958	0.424087	0.987951	0.041*	
H16B	0.170835	0.453916	1.074040	0.041*	
H16C	0.046744	0.396271	1.128112	0.041*	
Co1	0.48673 (4)	0.45310 (2)	0.88029 (3)	0.01554 (10)	
N1	0.38704 (19)	0.47189 (8)	0.70165 (14)	0.0156 (3)	
N2	0.2446 (2)	0.52316 (8)	0.54190 (14)	0.0172 (3)	
H2	0.191 (3)	0.5513 (13)	0.504 (2)	0.021*	
N3	0.7117 (2)	0.41951 (9)	0.82031 (15)	0.0208 (3)	
O1	0.43735 (17)	0.54921 (6)	0.92620 (12)	0.0176 (3)	
O2	0.30061 (18)	0.37837 (7)	0.89578 (13)	0.0216 (3)	
H2A	0.226 (4)	0.3794 (14)	0.836 (3)	0.032*	
S1	0.99084 (6)	0.38539 (2)	0.67772 (4)	0.01919 (12)	

### Bis[µ-2-(1H-1,3-benzodiazol-2-yl)phenolato]bis[ethanol(thiocyanato)cobalt(II)] (Struct-1) Atomic displacement parameters (Å^2^)

**Table:**

	*U*^11^	*U*^22^	*U*^33^	*U*^12^	*U*^13^	*U*^23^
C1	0.0154 (8)	0.0185 (8)	0.0126 (8)	−0.0021 (7)	0.0002 (6)	0.0019 (7)
C2	0.0166 (8)	0.0154 (8)	0.0164 (8)	−0.0003 (7)	0.0000 (7)	0.0007 (7)
C3	0.0166 (8)	0.0152 (8)	0.0162 (8)	−0.0002 (6)	0.0000 (7)	0.0018 (6)
C4	0.0212 (9)	0.0185 (9)	0.0172 (9)	−0.0007 (7)	−0.0031 (7)	−0.0008 (7)
C5	0.0256 (10)	0.0163 (9)	0.0219 (9)	0.0016 (7)	−0.0017 (7)	−0.0016 (7)
C6	0.0303 (10)	0.0183 (9)	0.0234 (10)	0.0054 (8)	−0.0046 (8)	0.0026 (7)
C7	0.0240 (9)	0.0204 (9)	0.0165 (9)	0.0033 (7)	−0.0042 (7)	0.0019 (7)
C8	0.0156 (8)	0.0187 (9)	0.0136 (8)	−0.0021 (7)	0.0016 (6)	−0.0006 (7)
C9	0.0190 (9)	0.0174 (9)	0.0151 (9)	−0.0020 (7)	0.0010 (7)	0.0006 (7)
C10	0.0294 (10)	0.0232 (10)	0.0133 (8)	−0.0015 (8)	−0.0019 (7)	0.0004 (7)
C11	0.0325 (11)	0.0232 (10)	0.0163 (9)	−0.0036 (8)	0.0006 (8)	−0.0044 (7)
C12	0.0258 (10)	0.0179 (9)	0.0217 (9)	0.0007 (7)	0.0024 (7)	−0.0030 (7)
C13	0.0206 (9)	0.0197 (9)	0.0189 (9)	0.0003 (7)	0.0001 (7)	0.0011 (7)
C14	0.0187 (9)	0.0169 (8)	0.0154 (8)	−0.0011 (7)	−0.0042 (7)	0.0021 (7)
C15	0.0244 (10)	0.0276 (10)	0.0192 (9)	−0.0038 (8)	0.0018 (7)	0.0051 (8)
C16	0.0238 (10)	0.0365 (12)	0.0213 (9)	−0.0016 (9)	−0.0011 (8)	−0.0031 (8)
Co1	0.01633 (16)	0.01559 (16)	0.01441 (16)	0.00107 (11)	−0.00140 (11)	−0.00038 (10)
N1	0.0162 (7)	0.0163 (7)	0.0141 (7)	0.0007 (6)	−0.0006 (6)	−0.0006 (6)
N2	0.0224 (8)	0.0152 (7)	0.0137 (7)	0.0009 (6)	−0.0029 (6)	0.0022 (6)
N3	0.0196 (8)	0.0241 (8)	0.0184 (8)	0.0017 (6)	−0.0019 (6)	0.0021 (6)
O1	0.0216 (7)	0.0160 (6)	0.0147 (6)	0.0018 (5)	−0.0031 (5)	0.0002 (5)
O2	0.0202 (7)	0.0285 (7)	0.0157 (6)	−0.0041 (6)	−0.0018 (5)	0.0016 (5)
S1	0.0169 (2)	0.0248 (2)	0.0158 (2)	−0.00064 (16)	−0.00012 (16)	0.00111 (16)

### Bis[µ-2-(1H-1,3-benzodiazol-2-yl)phenolato]bis[ethanol(thiocyanato)cobalt(II)] (Struct-1) Geometric parameters (Å, º)

**Table:**

C1—C2	1.460 (2)	C11—H11	0.9500
C1—N1	1.328 (2)	C11—C12	1.405 (3)
C1—N2	1.364 (2)	C12—H12	0.9500
C2—C3	1.418 (2)	C12—C13	1.387 (3)
C2—C7	1.410 (3)	C13—H13	0.9500
C3—C4	1.407 (3)	C14—N3	1.158 (3)
C3—O1	1.341 (2)	C14—S1	1.6402 (19)
C4—H4	0.9500	C15—H15A	0.9900
C4—C5	1.380 (3)	C15—H15B	0.9900
C5—H5	0.9500	C15—C16	1.499 (3)
C5—C6	1.394 (3)	C15—O2	1.451 (2)
C6—H6	0.9500	C16—H16A	0.9800
C6—C7	1.378 (3)	C16—H16B	0.9800
C7—H7	0.9500	C16—H16C	0.9800
C8—C9	1.396 (3)	Co1—N1	2.0509 (15)
C8—C13	1.396 (3)	Co1—N3	2.0002 (17)
C8—N1	1.393 (2)	Co1—O1^i^	2.1189 (13)
C9—C10	1.395 (3)	Co1—O1	1.9612 (13)
C9—N2	1.374 (2)	Co1—O2	2.0527 (14)
C10—H10	0.9500	N2—H2	0.78 (3)
C10—C11	1.384 (3)	O2—H2A	0.84 (3)
			
N1—C1—C2	126.68 (16)	C12—C13—H13	121.4
N1—C1—N2	110.54 (16)	N3—C14—S1	178.93 (18)
N2—C1—C2	122.77 (16)	H15A—C15—H15B	107.9
C3—C2—C1	122.87 (16)	C16—C15—H15A	109.3
C7—C2—C1	118.34 (16)	C16—C15—H15B	109.3
C7—C2—C3	118.79 (17)	O2—C15—H15A	109.3
C4—C3—C2	117.86 (16)	O2—C15—H15B	109.3
O1—C3—C2	123.68 (16)	O2—C15—C16	111.68 (17)
O1—C3—C4	118.47 (16)	C15—C16—H16A	109.5
C3—C4—H4	119.0	C15—C16—H16B	109.5
C5—C4—C3	122.09 (17)	C15—C16—H16C	109.5
C5—C4—H4	119.0	H16A—C16—H16B	109.5
C4—C5—H5	119.9	H16A—C16—H16C	109.5
C4—C5—C6	120.15 (18)	H16B—C16—H16C	109.5
C6—C5—H5	119.9	N1—Co1—O1^i^	169.03 (6)
C5—C6—H6	120.5	N1—Co1—O2	88.36 (6)
C7—C6—C5	119.01 (18)	N3—Co1—N1	92.62 (6)
C7—C6—H6	120.5	N3—Co1—O1^i^	96.29 (6)
C2—C7—H7	119.0	N3—Co1—O2	115.34 (6)
C6—C7—C2	122.09 (17)	O1—Co1—N1	89.89 (6)
C6—C7—H7	119.0	O1—Co1—N3	124.97 (6)
C9—C8—C13	120.67 (17)	O1—Co1—O1^i^	79.76 (5)
N1—C8—C9	108.59 (16)	O1—Co1—O2	119.68 (6)
N1—C8—C13	130.70 (17)	O2—Co1—O1^i^	93.57 (5)
C10—C9—C8	122.45 (17)	C1—N1—C8	106.74 (15)
N2—C9—C8	105.70 (16)	C1—N1—Co1	125.41 (12)
N2—C9—C10	131.81 (18)	C8—N1—Co1	127.66 (12)
C9—C10—H10	121.8	C1—N2—C9	108.43 (16)
C11—C10—C9	116.47 (18)	C1—N2—H2	125.3 (18)
C11—C10—H10	121.8	C9—N2—H2	126.2 (18)
C10—C11—H11	119.2	C14—N3—Co1	165.77 (15)
C10—C11—C12	121.63 (18)	C3—O1—Co1	130.96 (11)
C12—C11—H11	119.2	C3—O1—Co1^i^	127.89 (11)
C11—C12—H12	119.2	Co1—O1—Co1^i^	100.24 (5)
C13—C12—C11	121.55 (18)	C15—O2—Co1	128.51 (12)
C13—C12—H12	119.2	C15—O2—H2A	108.1 (19)
C8—C13—H13	121.4	Co1—O2—H2A	112.0 (19)
C12—C13—C8	117.23 (17)		
			
C1—C2—C3—C4	177.94 (17)	C9—C10—C11—C12	−0.3 (3)
C1—C2—C3—O1	−1.7 (3)	C10—C9—N2—C1	177.3 (2)
C1—C2—C7—C6	−178.60 (18)	C10—C11—C12—C13	0.7 (3)
C2—C1—N1—C8	−179.32 (17)	C11—C12—C13—C8	−0.6 (3)
C2—C1—N1—Co1	5.4 (3)	C13—C8—C9—C10	0.3 (3)
C2—C1—N2—C9	179.57 (16)	C13—C8—C9—N2	178.22 (16)
C2—C3—C4—C5	1.2 (3)	C13—C8—N1—C1	−177.78 (19)
C2—C3—O1—Co1	−3.7 (3)	C13—C8—N1—Co1	−2.6 (3)
C2—C3—O1—Co1^i^	−170.47 (13)	C16—C15—O2—Co1	67.7 (2)
C3—C2—C7—C6	0.6 (3)	N1—C1—C2—C3	0.5 (3)
C3—C4—C5—C6	−0.6 (3)	N1—C1—C2—C7	179.66 (18)
C4—C3—O1—Co1	176.65 (13)	N1—C1—N2—C9	0.3 (2)
C4—C3—O1—Co1^i^	9.8 (2)	N1—C8—C9—C10	−177.63 (18)
C4—C5—C6—C7	−0.1 (3)	N1—C8—C9—N2	0.3 (2)
C5—C6—C7—C2	0.1 (3)	N1—C8—C13—C12	177.49 (18)
C7—C2—C3—C4	−1.2 (3)	N2—C1—C2—C3	−178.64 (17)
C7—C2—C3—O1	179.14 (17)	N2—C1—C2—C7	0.5 (3)
C8—C9—C10—C11	−0.2 (3)	N2—C1—N1—C8	−0.1 (2)
C8—C9—N2—C1	−0.4 (2)	N2—C1—N1—Co1	−175.33 (12)
C9—C8—C13—C12	0.1 (3)	N2—C9—C10—C11	−177.55 (19)
C9—C8—N1—C1	−0.2 (2)	O1—C3—C4—C5	−179.09 (17)
C9—C8—N1—Co1	174.96 (12)		

Symmetry code: (i) −*x*+1, −*y*+1, −*z*+2.

### Bis[µ-2-(1H-1,3-benzodiazol-2-yl)phenolato]bis[ethanol(thiocyanato)cobalt(II)] (Struct-1) Hydrogen-bond geometry (Å, º)

**Table:**

*D*—H···*A*	*D*—H	H···*A*	*D*···*A*	*D*—H···*A*
N2—H2···S1^ii^	0.78 (3)	2.63 (3)	3.3843 (17)	162 (2)
O2—H2*A*···S1^iii^	0.84 (3)	2.42 (3)	3.2498 (15)	174 (2)

Symmetry codes: (ii) −*x*+1, −*y*+1, −*z*+1; (iii) *x*−1, *y*, *z*.

### Bis[µ-2-(1H-1,3-benzodiazol-2-yl)phenolato]bis{[2-(1H-1,3-benzodiazol-2-yl)phenolato](thiocyanato)manganese(III)} dihydrate (Struct-2) Crystal data

**Table:**

[Mn_2_(C_13_H_9_N_2_O)_4_(NCS)_2_]·2H_2_O	*F*(000) = 2256
*M**_r_* = 1098.96	*D*_x_ = 1.535 Mg m^−^^3^
Monoclinic, *C*2/*c*	Cu *K*α radiation, λ = 1.54178 Å
*a* = 21.8914 (6) Å	Cell parameters from 8754 reflections
*b* = 16.3110 (4) Å	θ = 3.4–74.0°
*c* = 13.5260 (4) Å	µ = 5.69 mm^−^^1^
β = 100.163 (2)°	*T* = 100 K
*V* = 4754.0 (2) Å^3^	(cut) plate, pale-brown
*Z* = 4	0.07 × 0.05 × 0.02 mm

### Bis[µ-2-(1H-1,3-benzodiazol-2-yl)phenolato]bis{[2-(1H-1,3-benzodiazol-2-yl)phenolato](thiocyanato)manganese(III)} dihydrate (Struct-2) Data collection

**Table:**

Rigaku 007HF diffractometer with HF Varimax confocal mirrors, an UG2 goniometer and HyPix Arc-100 detector	4776 independent reflections
Radiation source: Rotating anode, Rigaku 007 HF	3991 reflections with *I* > 2σ(*I*)
HF Varimax focusing mirrors monochromator	*R*_int_ = 0.043
Detector resolution: 10 pixels mm^-1^	θ_max_ = 74.5°, θ_min_ = 3.4°
profile data from ω–scans	*h* = −26→27
Absorption correction: gaussian (CrysAlisPro; Rigaku OD, 2023)	*k* = −20→16
*T*_min_ = 0.940, *T*_max_ = 1.000	*l* = −16→16
34537 measured reflections	

### Bis[µ-2-(1H-1,3-benzodiazol-2-yl)phenolato]bis{[2-(1H-1,3-benzodiazol-2-yl)phenolato](thiocyanato)manganese(III)} dihydrate (Struct-2) Refinement

**Table:**

Refinement on *F*^2^	Primary atom site location: dual
Least-squares matrix: full	Hydrogen site location: mixed
*R*[*F*^2^ > 2σ(*F*^2^)] = 0.039	H atoms treated by a mixture of independent and constrained refinement
*wR*(*F*^2^) = 0.107	*w* = 1/[σ^2^(*F*_o_^2^) + (0.0594*P*)^2^ + 4.7168*P*] where *P* = (*F*_o_^2^ + 2*F*_c_^2^)/3
*S* = 1.05	(Δ/σ)_max_ = 0.001
4776 reflections	Δρ_max_ = 1.24 e Å^−^^3^
346 parameters	Δρ_min_ = −0.48 e Å^−^^3^
2 restraints	

### Bis[µ-2-(1H-1,3-benzodiazol-2-yl)phenolato]bis{[2-(1H-1,3-benzodiazol-2-yl)phenolato](thiocyanato)manganese(III)} dihydrate (Struct-2) Special details

**Table:**

Geometry. All esds (except the esd in the dihedral angle between two l.s. planes) are estimated using the full covariance matrix. The cell esds are taken into account individually in the estimation of esds in distances, angles and torsion angles; correlations between esds in cell parameters are only used when they are defined by crystal symmetry. An approximate (isotropic) treatment of cell esds is used for estimating esds involving l.s. planes.
Refinement. The N-H bond lengths (N2-H2 and N4-H4a) have been restrained, otherwise they refined to unrealistic values. A peak of residual electron density (c.a. 1.2e-) at position (0.5, 0.264, 0.5 - close to atoms S1 and O3) remains unmodelled in the final refinement. During Structure development, a low occupancy water was modelled at this site (which also necessitated some disorder of the adjacent SCN ion). This arrangement gave rise to a chemically sensible hydrogen bond network and improved the refinement statistics; however, this arrangement freely refined to <8% occupancy and it has been omitted as this was deemed too low for reasonable certainty of the modelled disorder.

### Bis[µ-2-(1H-1,3-benzodiazol-2-yl)phenolato]bis{[2-(1H-1,3-benzodiazol-2-yl)phenolato](thiocyanato)manganese(III)} dihydrate (Struct-2) Fractional atomic coordinates and isotropic or equivalent isotropic displacement parameters (Å^2^)

**Table:**

	*x*	*y*	*z*	*U*_iso_*/*U*_eq_	
C1	0.45395 (11)	0.41180 (13)	0.62465 (15)	0.0255 (4)	
C2	0.39349 (11)	0.37490 (13)	0.62530 (16)	0.0262 (5)	
C3	0.38936 (10)	0.29465 (13)	0.66257 (16)	0.0254 (4)	
C4	0.33086 (11)	0.26158 (14)	0.66495 (17)	0.0289 (5)	
H4	0.327511	0.207719	0.690175	0.035*	
C5	0.27751 (11)	0.30661 (15)	0.63085 (18)	0.0340 (5)	
H5	0.237927	0.283114	0.631971	0.041*	
C6	0.28171 (12)	0.38602 (16)	0.59500 (19)	0.0361 (5)	
H6	0.245109	0.416890	0.572369	0.043*	
C7	0.33893 (11)	0.41961 (14)	0.59247 (17)	0.0318 (5)	
H7	0.341616	0.473887	0.568136	0.038*	
C8	0.55415 (11)	0.43002 (13)	0.63085 (16)	0.0269 (5)	
C9	0.52603 (12)	0.50736 (14)	0.62745 (16)	0.0283 (5)	
C10	0.56009 (12)	0.58000 (14)	0.63208 (17)	0.0335 (5)	
H10	0.540499	0.632110	0.629760	0.040*	
C11	0.62322 (13)	0.57260 (14)	0.64016 (18)	0.0363 (6)	
H11	0.647992	0.620678	0.643493	0.044*	
C12	0.65211 (12)	0.49509 (14)	0.64362 (17)	0.0330 (5)	
H12	0.695897	0.492175	0.648797	0.040*	
C13	0.61812 (11)	0.42302 (14)	0.63965 (17)	0.0298 (5)	
H13	0.637816	0.370946	0.642823	0.036*	
C14	0.56524 (10)	0.08065 (13)	0.63916 (15)	0.0231 (4)	
C15	0.62696 (10)	0.11510 (13)	0.64054 (16)	0.0254 (4)	
C16	0.63244 (10)	0.19544 (13)	0.60372 (16)	0.0266 (5)	
C17	0.69149 (11)	0.22342 (15)	0.59277 (17)	0.0312 (5)	
H17	0.695807	0.276429	0.565713	0.037*	
C18	0.74332 (11)	0.17511 (16)	0.62069 (17)	0.0334 (5)	
H18	0.782969	0.195372	0.613417	0.040*	
C19	0.73803 (11)	0.09661 (16)	0.65960 (18)	0.0336 (5)	
H19	0.773913	0.063791	0.679809	0.040*	
C20	0.68016 (11)	0.06732 (14)	0.66835 (17)	0.0301 (5)	
H20	0.676390	0.013613	0.693782	0.036*	
C21	0.46421 (10)	0.06703 (13)	0.61863 (15)	0.0238 (4)	
C22	0.48984 (10)	−0.01194 (13)	0.62563 (15)	0.0247 (4)	
C23	0.45339 (11)	−0.08204 (13)	0.61694 (16)	0.0271 (5)	
H23	0.471226	−0.135302	0.622449	0.032*	
C24	0.38993 (11)	−0.07056 (14)	0.59990 (16)	0.0288 (5)	
H24	0.363415	−0.117075	0.593848	0.035*	
C25	0.36361 (11)	0.00813 (14)	0.59129 (16)	0.0281 (5)	
H25	0.319720	0.013620	0.579369	0.034*	
C26	0.40020 (10)	0.07811 (13)	0.59978 (16)	0.0268 (5)	
H26	0.382304	0.131321	0.593015	0.032*	
C27	0.41872 (11)	0.21771 (13)	0.40867 (17)	0.0280 (5)	
Mn1	0.50948 (2)	0.24690 (2)	0.62879 (2)	0.02207 (11)	
N1	0.50728 (8)	0.37102 (11)	0.62871 (13)	0.0253 (4)	
N2	0.46335 (10)	0.49371 (11)	0.62275 (14)	0.0282 (4)	
H2	0.4364 (10)	0.5316 (14)	0.623 (2)	0.034*	
N3	0.51284 (8)	0.12376 (10)	0.62987 (12)	0.0231 (4)	
N4	0.55341 (9)	−0.00065 (11)	0.63934 (13)	0.0250 (4)	
H4A	0.5802 (10)	−0.0389 (13)	0.6434 (19)	0.030*	
N5	0.44756 (10)	0.23983 (11)	0.48419 (15)	0.0309 (4)	
O1	0.44065 (7)	0.25005 (8)	0.69943 (11)	0.0240 (3)	
O2	0.58367 (8)	0.24520 (8)	0.57549 (12)	0.0275 (3)	
O3	0.59279 (9)	0.34515 (12)	0.39364 (16)	0.0419 (4)	
H3A	0.5869 (17)	0.319 (2)	0.444 (3)	0.063*	
H3B	0.5904 (16)	0.310 (2)	0.343 (3)	0.063*	
S1	0.37974 (3)	0.18737 (4)	0.30045 (5)	0.03899 (17)	

### Bis[µ-2-(1H-1,3-benzodiazol-2-yl)phenolato]bis{[2-(1H-1,3-benzodiazol-2-yl)phenolato](thiocyanato)manganese(III)} dihydrate (Struct-2) Atomic displacement parameters (Å^2^)

**Table:**

	*U*^11^	*U*^22^	*U*^33^	*U*^12^	*U*^13^	*U*^23^
C1	0.0363 (12)	0.0218 (10)	0.0181 (10)	0.0027 (9)	0.0041 (8)	0.0014 (8)
C2	0.0338 (12)	0.0237 (11)	0.0215 (10)	0.0026 (9)	0.0060 (9)	−0.0008 (8)
C3	0.0311 (11)	0.0234 (10)	0.0220 (10)	0.0047 (9)	0.0054 (8)	−0.0011 (8)
C4	0.0328 (12)	0.0275 (11)	0.0264 (11)	0.0011 (9)	0.0058 (9)	0.0000 (9)
C5	0.0300 (12)	0.0369 (13)	0.0350 (13)	0.0032 (10)	0.0052 (10)	−0.0008 (10)
C6	0.0345 (13)	0.0376 (13)	0.0349 (13)	0.0110 (11)	0.0027 (10)	0.0016 (10)
C7	0.0414 (13)	0.0273 (11)	0.0265 (11)	0.0088 (10)	0.0058 (10)	0.0033 (9)
C8	0.0400 (13)	0.0207 (10)	0.0207 (10)	−0.0045 (9)	0.0069 (9)	0.0018 (8)
C9	0.0429 (13)	0.0233 (11)	0.0187 (10)	−0.0010 (10)	0.0051 (9)	0.0017 (8)
C10	0.0488 (15)	0.0201 (11)	0.0318 (12)	−0.0028 (10)	0.0073 (11)	0.0038 (9)
C11	0.0505 (15)	0.0259 (11)	0.0323 (12)	−0.0111 (11)	0.0072 (11)	0.0016 (10)
C12	0.0400 (13)	0.0300 (12)	0.0294 (12)	−0.0075 (10)	0.0074 (10)	0.0005 (9)
C13	0.0383 (13)	0.0257 (11)	0.0260 (11)	−0.0028 (10)	0.0078 (9)	0.0004 (9)
C14	0.0306 (11)	0.0216 (10)	0.0172 (9)	0.0018 (9)	0.0048 (8)	0.0007 (8)
C15	0.0313 (11)	0.0252 (10)	0.0205 (10)	0.0005 (9)	0.0064 (8)	−0.0028 (8)
C16	0.0319 (12)	0.0256 (11)	0.0235 (10)	−0.0010 (9)	0.0078 (9)	−0.0024 (8)
C17	0.0361 (13)	0.0292 (11)	0.0295 (12)	−0.0025 (10)	0.0089 (10)	−0.0011 (9)
C18	0.0311 (12)	0.0381 (13)	0.0326 (12)	−0.0046 (10)	0.0101 (10)	−0.0028 (10)
C19	0.0313 (12)	0.0379 (13)	0.0313 (12)	0.0047 (10)	0.0050 (9)	−0.0010 (10)
C20	0.0350 (12)	0.0271 (11)	0.0284 (11)	0.0018 (10)	0.0058 (9)	0.0000 (9)
C21	0.0333 (12)	0.0197 (10)	0.0193 (10)	−0.0023 (9)	0.0073 (8)	−0.0001 (8)
C22	0.0323 (12)	0.0235 (10)	0.0186 (10)	0.0013 (9)	0.0053 (8)	0.0008 (8)
C23	0.0372 (12)	0.0199 (10)	0.0248 (11)	−0.0014 (9)	0.0074 (9)	−0.0004 (8)
C24	0.0388 (13)	0.0266 (11)	0.0220 (11)	−0.0076 (10)	0.0080 (9)	−0.0027 (8)
C25	0.0300 (12)	0.0299 (11)	0.0246 (11)	−0.0022 (9)	0.0055 (9)	−0.0021 (9)
C26	0.0323 (12)	0.0233 (11)	0.0251 (11)	0.0007 (9)	0.0056 (9)	−0.0007 (8)
C27	0.0311 (12)	0.0204 (10)	0.0333 (13)	0.0039 (9)	0.0082 (10)	−0.0012 (9)
Mn1	0.02793 (19)	0.01535 (18)	0.02377 (19)	0.00071 (13)	0.00685 (14)	0.00080 (12)
N1	0.0340 (10)	0.0187 (9)	0.0239 (9)	0.0003 (7)	0.0067 (8)	0.0021 (7)
N2	0.0403 (11)	0.0185 (9)	0.0264 (9)	0.0031 (8)	0.0072 (8)	0.0025 (7)
N3	0.0298 (10)	0.0181 (8)	0.0217 (9)	−0.0011 (7)	0.0054 (7)	−0.0004 (6)
N4	0.0315 (10)	0.0192 (9)	0.0248 (9)	0.0021 (7)	0.0065 (8)	−0.0001 (7)
N5	0.0400 (11)	0.0235 (9)	0.0283 (10)	0.0036 (8)	0.0037 (9)	0.0030 (8)
O1	0.0281 (8)	0.0200 (7)	0.0238 (8)	0.0007 (6)	0.0038 (6)	0.0022 (5)
O2	0.0325 (9)	0.0217 (8)	0.0295 (8)	0.0009 (6)	0.0085 (7)	0.0035 (6)
O3	0.0521 (11)	0.0348 (10)	0.0406 (11)	0.0063 (8)	0.0130 (9)	0.0089 (8)
S1	0.0398 (3)	0.0360 (3)	0.0373 (3)	0.0049 (3)	−0.0040 (2)	−0.0100 (3)

### Bis[µ-2-(1H-1,3-benzodiazol-2-yl)phenolato]bis{[2-(1H-1,3-benzodiazol-2-yl)phenolato](thiocyanato)manganese(III)} dihydrate (Struct-2) Geometric parameters (Å, º)

**Table:**

C1—C2	1.455 (3)	C16—O2	1.342 (3)
C1—N1	1.336 (3)	C17—H17	0.9500
C1—N2	1.353 (3)	C17—C18	1.378 (4)
C2—C3	1.411 (3)	C18—H18	0.9500
C2—C7	1.403 (3)	C18—C19	1.397 (4)
C3—C4	1.395 (3)	C19—H19	0.9500
C3—O1	1.357 (3)	C19—C20	1.379 (3)
C4—H4	0.9500	C20—H20	0.9500
C4—C5	1.388 (3)	C21—C22	1.402 (3)
C5—H5	0.9500	C21—C26	1.391 (3)
C5—C6	1.392 (4)	C21—N3	1.398 (3)
C6—H6	0.9500	C22—C23	1.387 (3)
C6—C7	1.373 (4)	C22—N4	1.383 (3)
C7—H7	0.9500	C23—H23	0.9500
C8—C9	1.401 (3)	C23—C24	1.380 (3)
C8—C13	1.389 (3)	C24—H24	0.9500
C8—N1	1.403 (3)	C24—C25	1.403 (3)
C9—C10	1.395 (3)	C25—H25	0.9500
C9—N2	1.380 (3)	C25—C26	1.388 (3)
C10—H10	0.9500	C26—H26	0.9500
C10—C11	1.372 (4)	C27—N5	1.160 (3)
C11—H11	0.9500	C27—S1	1.636 (2)
C11—C12	1.411 (4)	Mn1—N1	2.0251 (18)
C12—H12	0.9500	Mn1—N3	2.0099 (17)
C12—C13	1.387 (3)	Mn1—N5	2.177 (2)
C13—H13	0.9500	Mn1—O1^i^	2.3869 (16)
C14—C15	1.460 (3)	Mn1—O1	1.9219 (16)
C14—N3	1.332 (3)	Mn1—O2	1.8898 (17)
C14—N4	1.351 (3)	N2—H2	0.854 (17)
C15—C16	1.415 (3)	N4—H4A	0.851 (17)
C15—C20	1.397 (3)	O3—H3A	0.83 (4)
C16—C17	1.403 (3)	O3—H3B	0.89 (4)
			
N1—C1—C2	125.64 (19)	C20—C19—H19	120.4
N1—C1—N2	111.0 (2)	C15—C20—H20	119.4
N2—C1—C2	123.4 (2)	C19—C20—C15	121.2 (2)
C3—C2—C1	120.1 (2)	C19—C20—H20	119.4
C7—C2—C1	120.5 (2)	C26—C21—C22	120.7 (2)
C7—C2—C3	119.4 (2)	C26—C21—N3	131.1 (2)
C4—C3—C2	118.9 (2)	N3—C21—C22	108.22 (19)
O1—C3—C2	121.8 (2)	C23—C22—C21	122.3 (2)
O1—C3—C4	119.20 (19)	N4—C22—C21	105.55 (18)
C3—C4—H4	119.7	N4—C22—C23	132.1 (2)
C5—C4—C3	120.7 (2)	C22—C23—H23	121.7
C5—C4—H4	119.7	C24—C23—C22	116.7 (2)
C4—C5—H5	119.8	C24—C23—H23	121.7
C4—C5—C6	120.3 (2)	C23—C24—H24	119.2
C6—C5—H5	119.8	C23—C24—C25	121.6 (2)
C5—C6—H6	120.1	C25—C24—H24	119.2
C7—C6—C5	119.8 (2)	C24—C25—H25	119.2
C7—C6—H6	120.1	C26—C25—C24	121.5 (2)
C2—C7—H7	119.5	C26—C25—H25	119.2
C6—C7—C2	120.9 (2)	C21—C26—H26	121.4
C6—C7—H7	119.5	C25—C26—C21	117.2 (2)
C9—C8—N1	107.5 (2)	C25—C26—H26	121.4
C13—C8—C9	120.5 (2)	N5—C27—S1	178.3 (2)
C13—C8—N1	131.9 (2)	N1—Mn1—N5	92.38 (7)
C10—C9—C8	122.3 (2)	N1—Mn1—O1^i^	89.18 (6)
N2—C9—C8	106.49 (19)	N3—Mn1—N1	179.23 (8)
N2—C9—C10	131.1 (2)	N3—Mn1—N5	88.27 (7)
C9—C10—H10	121.6	N3—Mn1—O1^i^	90.26 (6)
C11—C10—C9	116.8 (2)	N5—Mn1—O1^i^	168.77 (7)
C11—C10—H10	121.6	O1—Mn1—N1	87.30 (7)
C10—C11—H11	119.3	O1—Mn1—N3	93.10 (6)
C10—C11—C12	121.4 (2)	O1—Mn1—N5	91.70 (7)
C12—C11—H11	119.3	O1—Mn1—O1^i^	77.26 (7)
C11—C12—H12	119.2	O2—Mn1—N1	92.09 (7)
C13—C12—C11	121.6 (2)	O2—Mn1—N3	87.43 (7)
C13—C12—H12	119.2	O2—Mn1—N5	95.58 (8)
C8—C13—H13	121.3	O2—Mn1—O1	172.72 (7)
C12—C13—C8	117.3 (2)	O2—Mn1—O1^i^	95.48 (6)
C12—C13—H13	121.3	C1—N1—C8	106.84 (18)
N3—C14—C15	125.27 (19)	C1—N1—Mn1	121.21 (15)
N3—C14—N4	110.85 (19)	C8—N1—Mn1	131.94 (15)
N4—C14—C15	123.69 (19)	C1—N2—C9	108.16 (19)
C16—C15—C14	119.19 (19)	C1—N2—H2	127.4 (19)
C20—C15—C14	120.96 (19)	C9—N2—H2	124.4 (19)
C20—C15—C16	119.6 (2)	C14—N3—C21	106.70 (17)
C17—C16—C15	118.4 (2)	C14—N3—Mn1	123.91 (14)
O2—C16—C15	123.2 (2)	C21—N3—Mn1	129.36 (14)
O2—C16—C17	118.3 (2)	C14—N4—C22	108.60 (18)
C16—C17—H17	119.5	C14—N4—H4A	126.1 (18)
C18—C17—C16	120.9 (2)	C22—N4—H4A	125.2 (18)
C18—C17—H17	119.5	C27—N5—Mn1	164.52 (17)
C17—C18—H18	119.7	C3—O1—Mn1	120.15 (13)
C17—C18—C19	120.6 (2)	C3—O1—Mn1^i^	125.16 (12)
C19—C18—H18	119.7	Mn1—O1—Mn1^i^	102.67 (7)
C18—C19—H19	120.4	C16—O2—Mn1	126.48 (13)
C20—C19—C18	119.2 (2)	H3A—O3—H3B	108 (3)
			
C1—C2—C3—C4	178.2 (2)	C20—C15—C16—C17	2.4 (3)
C1—C2—C3—O1	0.7 (3)	C20—C15—C16—O2	−180.0 (2)
C1—C2—C7—C6	−178.5 (2)	C21—C22—C23—C24	0.6 (3)
C2—C1—N1—C8	−177.4 (2)	C21—C22—N4—C14	−0.8 (2)
C2—C1—N1—Mn1	3.5 (3)	C22—C21—C26—C25	1.8 (3)
C2—C1—N2—C9	177.21 (19)	C22—C21—N3—C14	2.3 (2)
C2—C3—C4—C5	0.3 (3)	C22—C21—N3—Mn1	−179.52 (14)
C2—C3—O1—Mn1^i^	91.4 (2)	C22—C23—C24—C25	0.3 (3)
C2—C3—O1—Mn1	−44.6 (2)	C23—C22—N4—C14	177.7 (2)
C3—C2—C7—C6	−0.8 (3)	C23—C24—C25—C26	−0.2 (3)
C3—C4—C5—C6	−0.9 (4)	C24—C25—C26—C21	−0.9 (3)
C4—C3—O1—Mn1	137.83 (17)	C26—C21—C22—C23	−1.7 (3)
C4—C3—O1—Mn1^i^	−86.2 (2)	C26—C21—C22—N4	177.00 (18)
C4—C5—C6—C7	0.7 (4)	C26—C21—N3—C14	−175.3 (2)
C5—C6—C7—C2	0.2 (4)	C26—C21—N3—Mn1	2.8 (3)
C7—C2—C3—C4	0.5 (3)	N1—C1—C2—C3	20.7 (3)
C7—C2—C3—O1	−177.02 (19)	N1—C1—C2—C7	−161.7 (2)
C8—C9—C10—C11	0.0 (3)	N1—C1—N2—C9	−1.0 (2)
C8—C9—N2—C1	0.8 (2)	N1—C8—C9—C10	177.6 (2)
C9—C8—C13—C12	−0.7 (3)	N1—C8—C9—N2	−0.4 (2)
C9—C8—N1—C1	−0.2 (2)	N1—C8—C13—C12	−177.3 (2)
C9—C8—N1—Mn1	178.78 (15)	N1—Mn1—O2—C16	138.39 (17)
C9—C10—C11—C12	0.0 (4)	N2—C1—C2—C3	−157.3 (2)
C10—C9—N2—C1	−176.9 (2)	N2—C1—C2—C7	20.4 (3)
C10—C11—C12—C13	−0.4 (4)	N2—C1—N1—C8	0.8 (2)
C11—C12—C13—C8	0.8 (3)	N2—C1—N1—Mn1	−178.37 (13)
C13—C8—C9—C10	0.3 (3)	N2—C9—C10—C11	177.5 (2)
C13—C8—C9—N2	−177.64 (19)	N3—C14—C15—C16	−17.8 (3)
C13—C8—N1—C1	176.6 (2)	N3—C14—C15—C20	168.5 (2)
C13—C8—N1—Mn1	−4.4 (4)	N3—C14—N4—C22	2.4 (2)
C14—C15—C16—C17	−171.4 (2)	N3—C21—C22—C23	−179.65 (19)
C14—C15—C16—O2	6.2 (3)	N3—C21—C22—N4	−0.9 (2)
C14—C15—C20—C19	173.0 (2)	N3—C21—C26—C25	179.2 (2)
C15—C14—N3—C21	172.27 (19)	N3—Mn1—O2—C16	−41.01 (17)
C15—C14—N3—Mn1	−6.0 (3)	N4—C14—C15—C16	156.8 (2)
C15—C14—N4—C22	−172.89 (19)	N4—C14—C15—C20	−17.0 (3)
C15—C16—C17—C18	−2.4 (3)	N4—C14—N3—C21	−2.9 (2)
C15—C16—O2—Mn1	30.7 (3)	N4—C14—N3—Mn1	178.83 (13)
C16—C15—C20—C19	−0.7 (3)	N4—C22—C23—C24	−177.7 (2)
C16—C17—C18—C19	0.7 (4)	N5—Mn1—O2—C16	−129.02 (17)
C17—C16—O2—Mn1	−151.68 (16)	O1—C3—C4—C5	177.9 (2)
C17—C18—C19—C20	1.0 (4)	O1^i^—Mn1—O2—C16	49.01 (17)
C18—C19—C20—C15	−1.0 (4)	O2—C16—C17—C18	179.8 (2)

Symmetry code: (i) −*x*+1, *y*, −*z*+3/2.

### Bis[µ-2-(1H-1,3-benzodiazol-2-yl)phenolato]bis{[2-(1H-1,3-benzodiazol-2-yl)phenolato](thiocyanato)manganese(III)} dihydrate (Struct-2) Hydrogen-bond geometry (Å, º)

**Table:**

*D*—H···*A*	*D*—H	H···*A*	*D*···*A*	*D*—H···*A*
N2—H2···O3^ii^	0.85 (2)	2.11 (2)	2.893 (3)	152 (2)
N4—H4*A*···S1^iii^	0.85 (2)	2.64 (2)	3.4147 (19)	152 (2)
O3—H3*A*···O2	0.83 (4)	2.17 (4)	2.987 (2)	173 (3)
O3—H3*B*···S1^iv^	0.89 (4)	2.94 (4)	3.799 (2)	163 (3)

Symmetry codes: (ii) −*x*+1, −*y*+1, −*z*+1; (iii) −*x*+1, −*y*, −*z*+1; (iv) −*x*+1, *y*, −*z*+1/2.
