# Supplementary material for: Synthesis and crystal structures of two related Co and Mn complexes: a celebration of collaboration between the universities of Dakar and Southampton
Source: Acta Crystallogr E Crystallogr Commun. 2023 Nov 16;79(Pt 12):1109–14. doi: 10.1107/S2056989023009805 (PMC10833403; doi:10.1107/S2056989023009805)

Supporting Information

SI-1: Distance and Angle Comparison of hydrogen bonds within **1** (CSD-Materials/Mercury; CSD v5.44, Apr 2023)

**Donor**	**Acceptor**	**Distance D-A**	**Distance classification**	**Distance threshold**	**Distance hits**	**Distance mean**	**Distance std. dev.**	**Distance min.**	**Distance max.**	**Angle D-H…A**	**Angle classification**	**Angle threshold**	**Angle hits**	**Angle mean**	**Angle std. dev.**	**Angle min.**	**Angle max.**
N2 (amine)	S1 [1] (CdoubleS)	**3.38**	**Not Unusual**	(3.38, 3.49)	2	3.43	0.08	3.38	3.49	**161.69**	**Not Unusual**	123.81	2	135.46	18.31	122.52	148.41
O2 (al_hydroxy)	S1 [2] (CdoubleS)	**3.25**	**Not Unusual**	(3.19, 3.51)	87	3.29	0.13	3.11	3.77	**174.54**	**Not Unusual**	132.65	87	166.51	15.35	122.51	179.39

[1]1-x,1-y,1-z; [2]-1+x,+y,+z

N2-H2···O3: | Distance | Angle | Frequency | to similar examples on the CSD

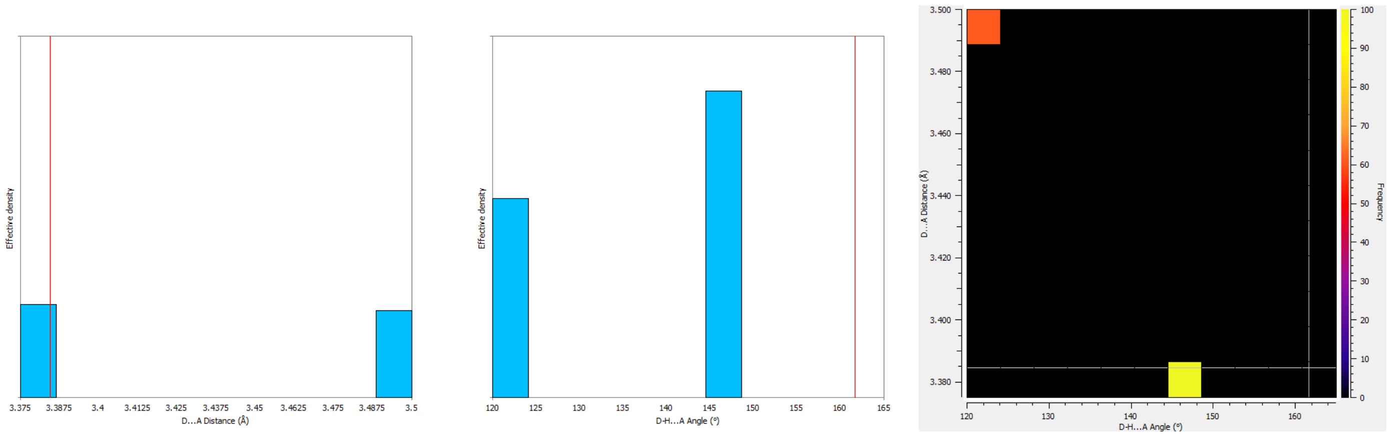

O2-H2a···O3: | Distance | Angle | Frequency | to similar examples on the CSD

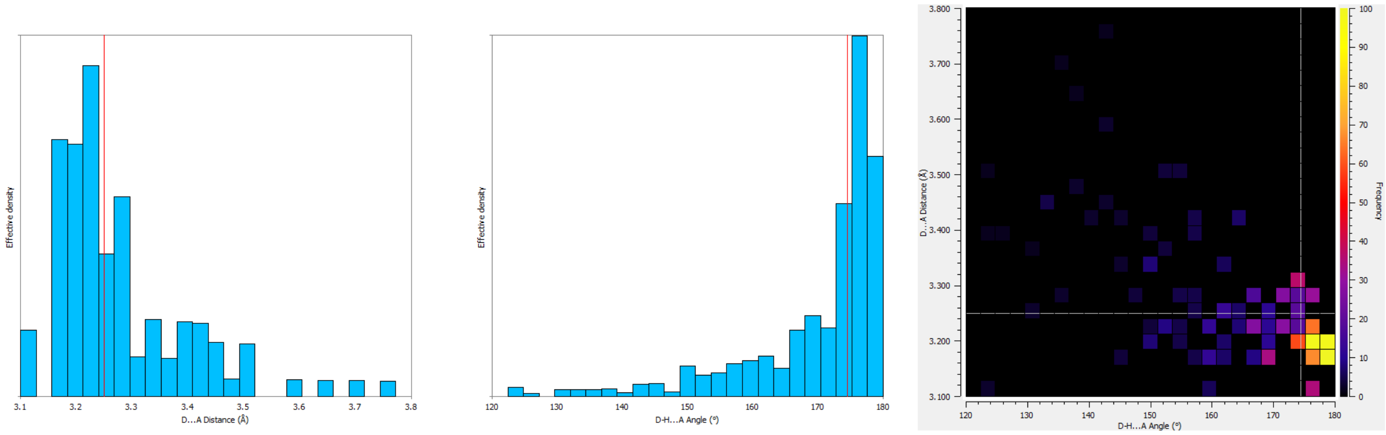

SI-2: Distance and Angle Comparison of hydrogen bonds within **2** (CSD-Materials/Mercury; CSD v5.44, Apr 2023)

**Donor**	**Acceptor**	**Distance D-A**	**Distance classification**	**Distance threshold**	**Distance hits**	**Distance mean**	**Distance std. dev.**	**Distance min.**	**Distance max.**	**Angle D-H...A**	**Angle classification**	**Angle threshold**	**Angle hits**	**Angle mean**	**Angle std. dev.**	**Angle min.**	**Angle max.**
N2 (amine_5_1)	O3 (water)	**2.89**	**Not Unusual**	(2.71, 3.02)	132	2.82	0.11	2.63	3.25	**152.24**	**Not Unusual**	151.36	132	170.29	10.1	120.37	179.21
O3 (water)	O2 (T2OH0)	**2.99**	**Not Unusual**	(2.77, 3.37)	2092	2.99	0.19	2.64	3.79	**172.58**	**Not Unusual**	134.48	2092	168.41	13.85	120.03	179.96
N4 (amine_5_1)	S1 (CdoubleS)	**3.41**	**Not Unusual**	(3.38, 3.49)	2	3.43	0.08	3.38	3.49	**151.7**	**Not Unusual**	123.81	2	135.46	18.31	122.52	148.41
O3 (water)	S1 (CdoubleS)	**3.8**	**Unusual**	(3.15, 3.57)	224	3.33	0.11	2.93	3.71	**163.57**	**Not Unusual**	139.8	224	169.01	12.23	122.5	179.61

N2-H2···O3: | Distance | Angle | Frequency | to similar examples on the CSD

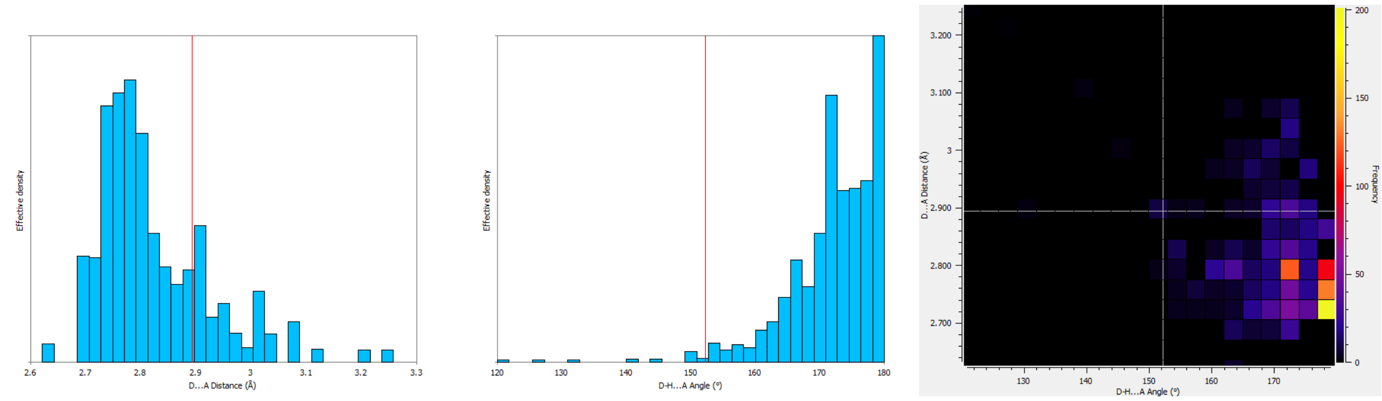

O3-H3a···O2: | Distance | Angle | Frequency | to similar examples on the CSD

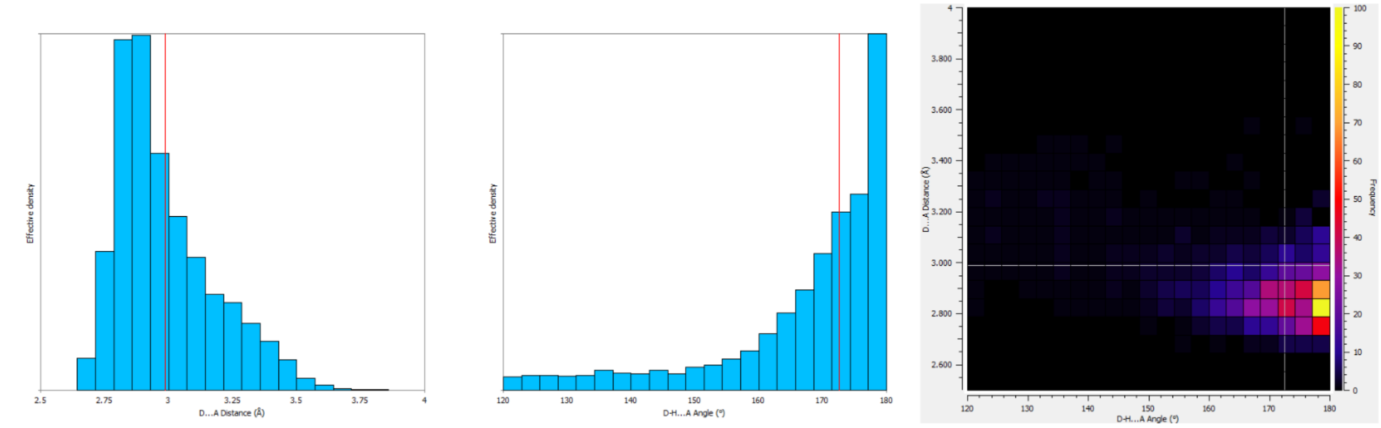

N4-H4a···S1: | Distance | Angle | Frequency | to similar examples on the CSD

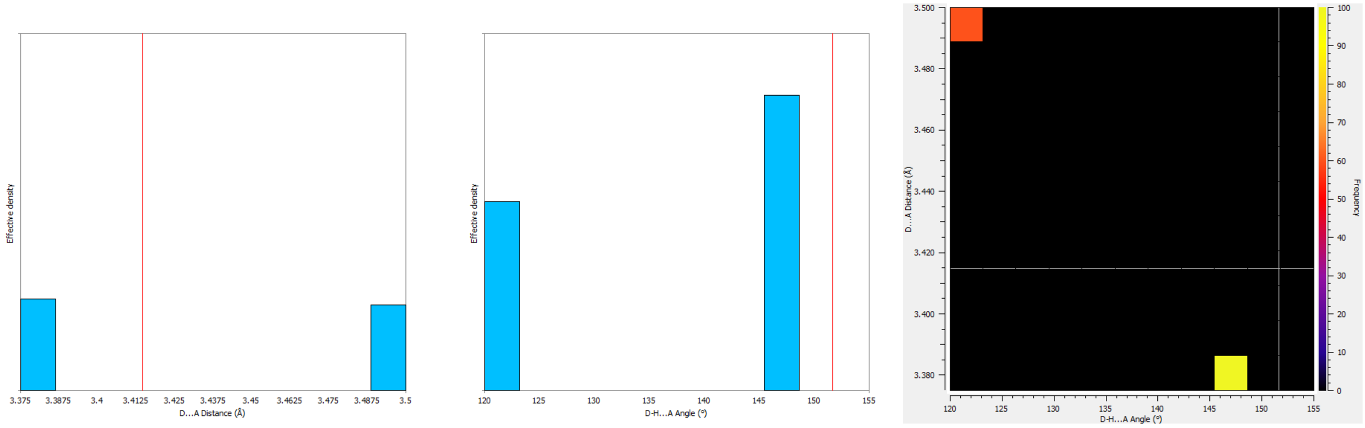

O3-H3b···S1: | Distance | Angle | Frequency | to similar examples on the CSD

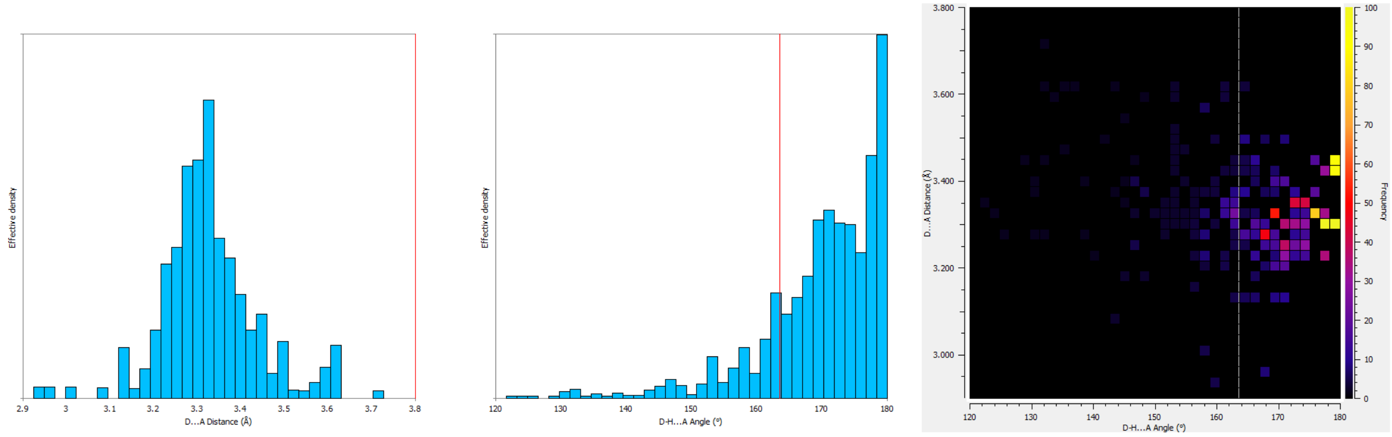

Views of Complex 2 highlighting hydrogen bonding (thermal ellipsoids drawn at 50% probability)

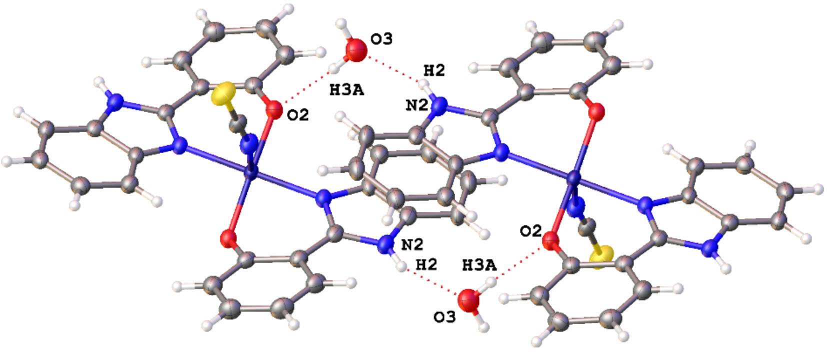

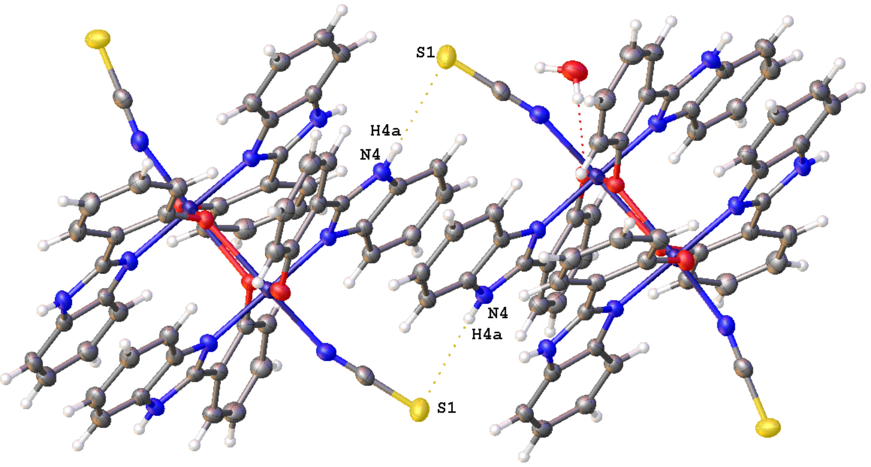

SI-3: Spectroscopic information

^{1}H and ^{13}C NMR acquired in DMSO-d_6 or $CDCl_3$ on BRUKER AVANCE 500 spectrometer, using TMS as an internal standard. UV-vis spectra recorded using a SCINCO S-3100 spectrophotometer with a photodiode (PDA) detector. FT-IR spectra obtained upon a PerkinElmer Spectrum Two instrument (DTGS). Molar conductivity measurements performed using an EC-214 conductivity meter at at room temperature and using a cell containing a millimolar solution of dimethylformamide.

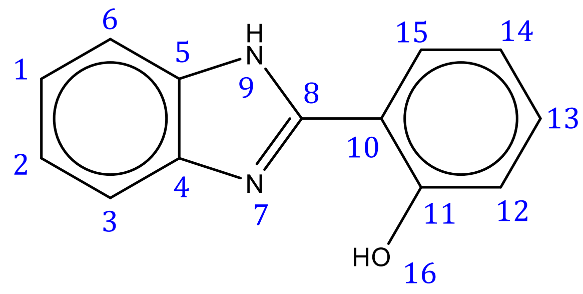

2hpbi ligand

Infrared spectroscopy: 2hpbi ligand, (2-(1H-benzimidazol-2-yl)phenol ($C_{13}H_{10}N_2O$)

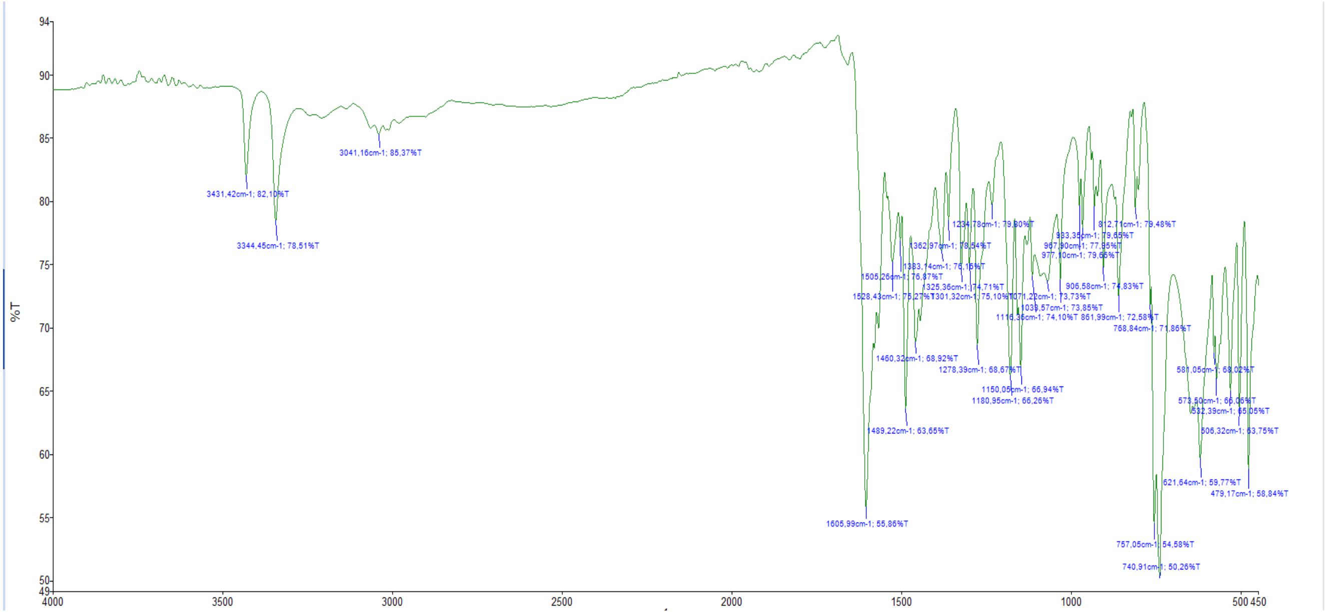

Infrared spectroscopy: Complex **1**, $[Co_2(C_{13}H_9N_2O)_2(SCN)_2(CH_3CH_2OH)_2]$

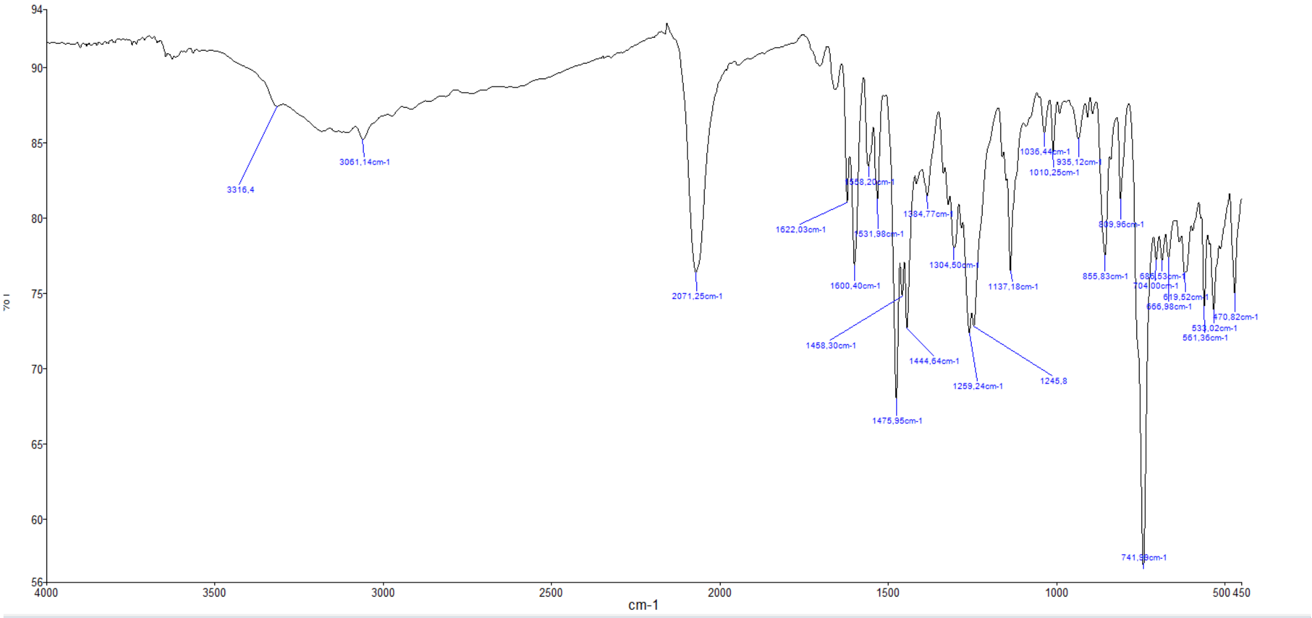

Infrared spectroscopy: Complex **2** $[Mn_2(C_{13}H_9N_2O)_4(SCN)_2].2(H_2O)$

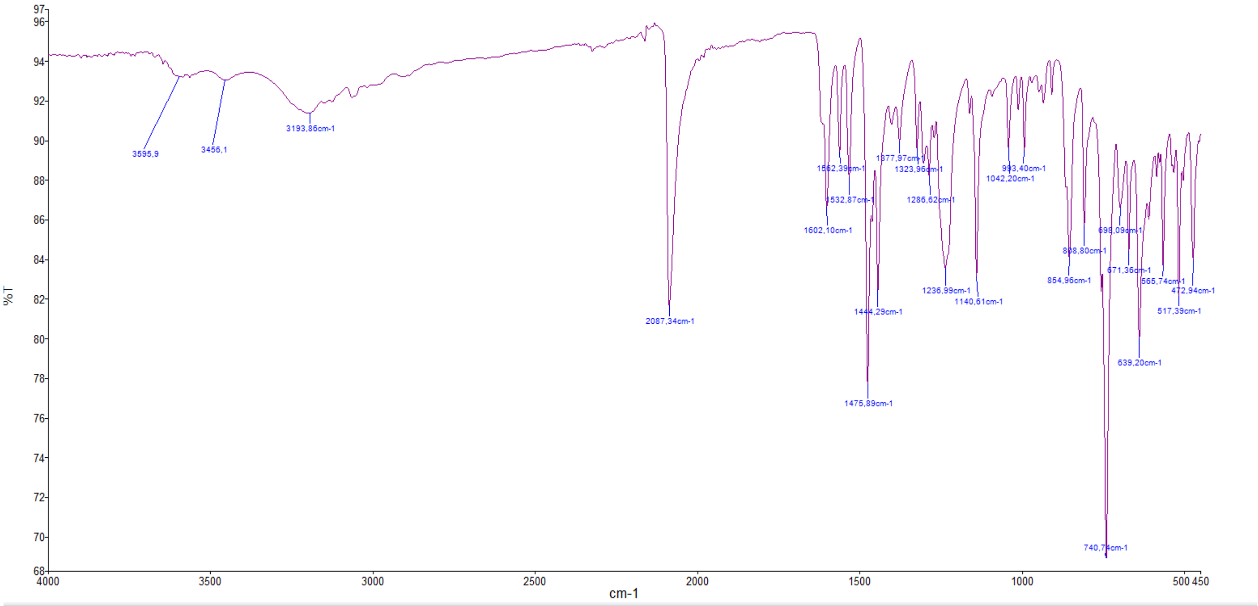

^{1}H NMR: 2hpbi ligand, (2-(1H-benzimidazol-2-yl)phenol ($C_{13}H_{10}N_2O$)

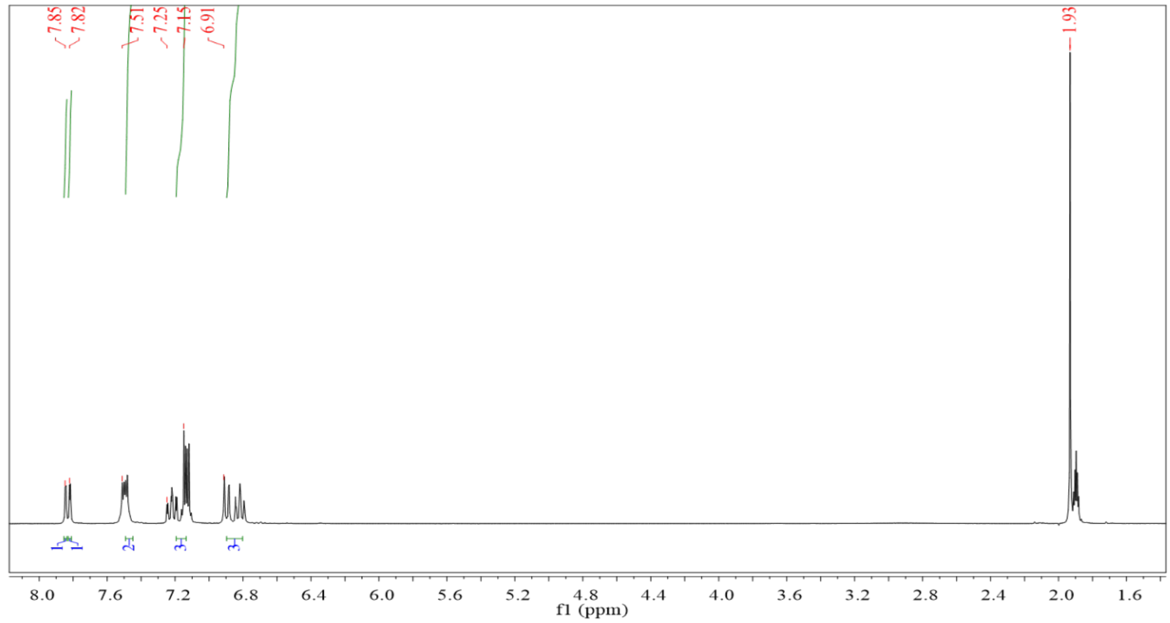

^{13}C NMR: 2hpbi ligand, (2-(1H-benzimidazol-2-yl)phenol ($C_{13}H_{10}N_2O$)

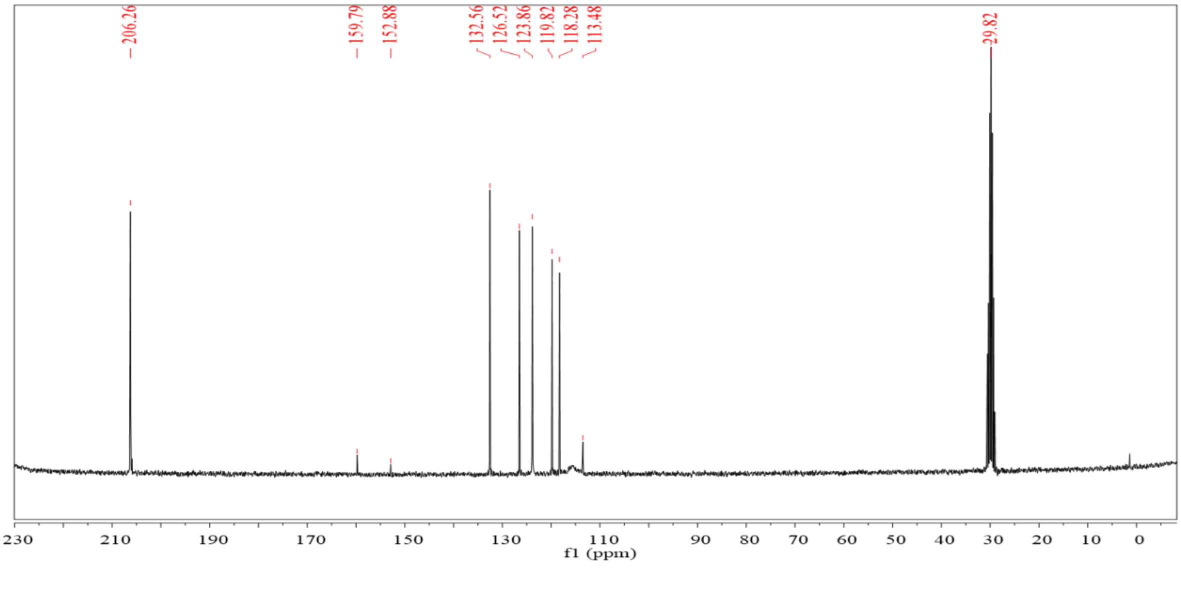

^{13}C NMR (DEPT 135): 2hpbi ligand, (2-(1H-benzimidazol-2-yl)phenol ($C_{13}H_{10}N_2O$)

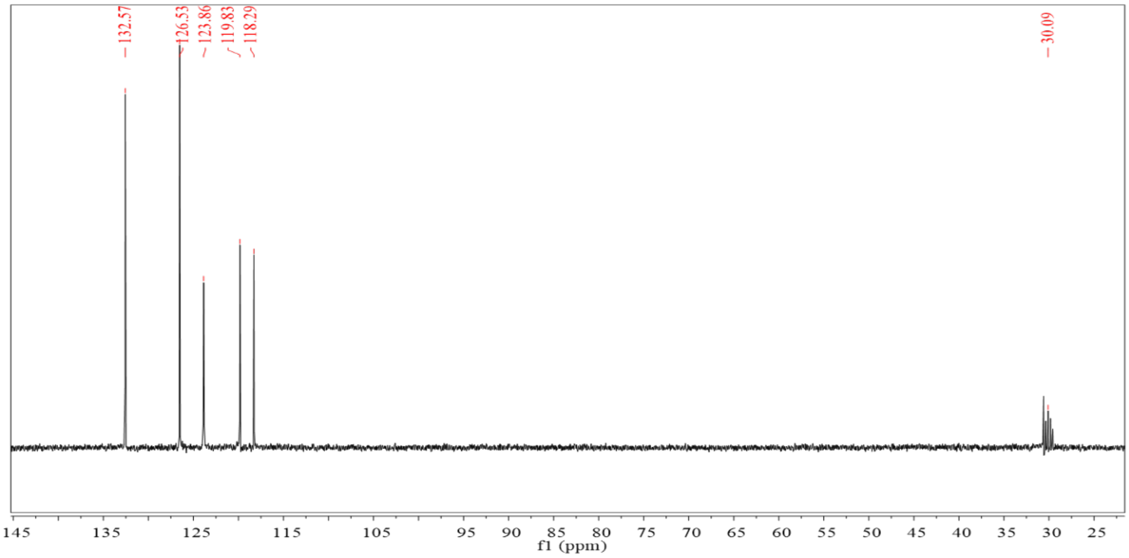

Conductivity study: Complexes **1** and **2**

	**Cold Conductivity** (µS/cm)	**Conductivity after 15 days** (µS/cm)	**Electrolytes**
**Complex 1**	19,26	17,21	Neutral
**Complex 2**	18,44	16,95	Neutral

UV-Vis Spectroscopy: Complex **1**, [$Co_2(C_{13}H_9N_2O)_2(SCN)_2(CH_3CH_2OH)_2$]

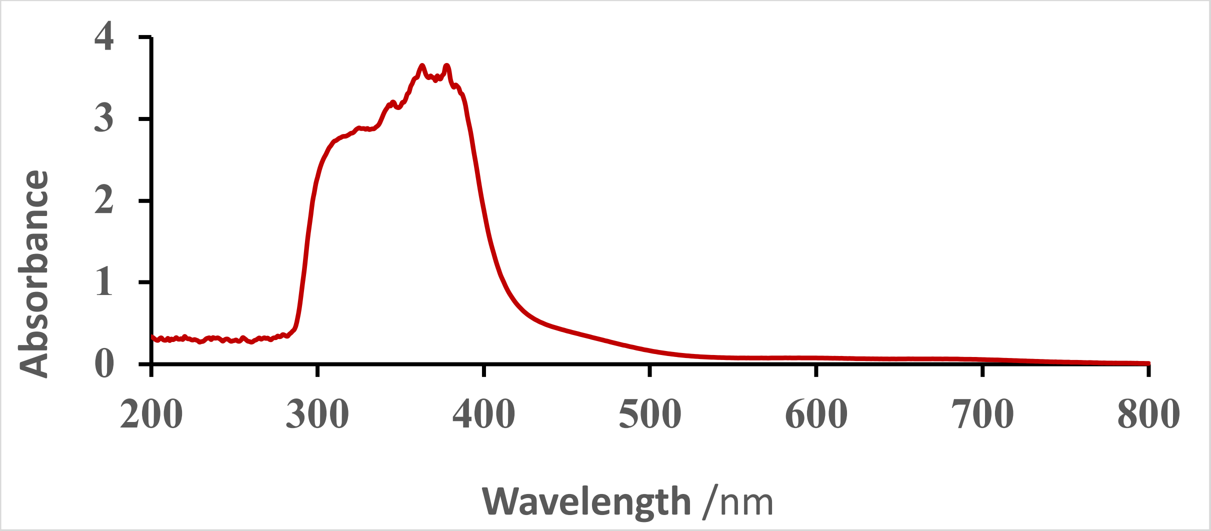

UV-Vis Spectroscopy: Complex **2** [$Mn_2(C_{13}H_9N_2O)_4(SCN)_2$].2($H_2O$)

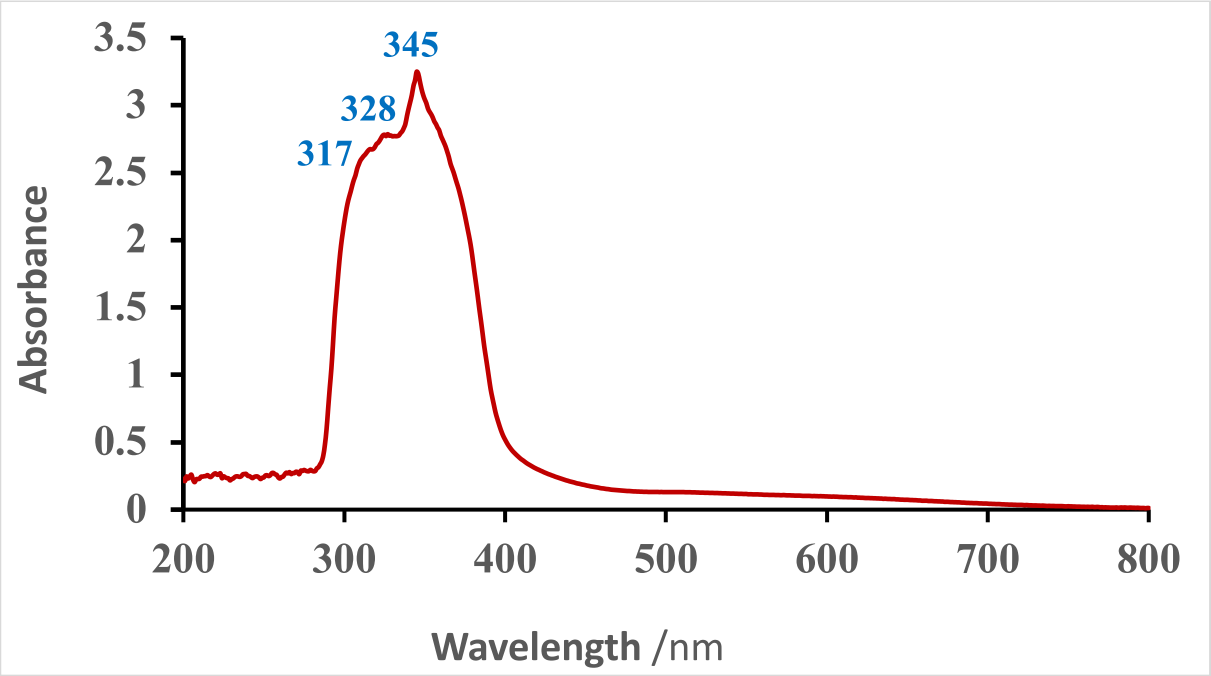

SI-4: Complex **1** coordination polyhedron geometry index

The geometry around the penta-coordinated cobalt (II) ion is deduced from the trigonality or Addison index. This is defined by the relation **$\tau = (\beta - \alpha)/60$** which is applicable to five-co-ordinate structures as an index of the degree of trigonality. β and α are the greatest angles of connection around Co(II). If $\tau = 0$ the geometry is a perfect square pyramid while a value of $\tau = 1$ refers to a trigonal bipyramidal geometry. The greatest angles around Co(II) are β=169.020° for N1-Co1-O1^{i} and α=119.680° for O2-Co-O1. The value thus found is $\tau = 0.82$ confirms a slightly deformed trigonal-bipyramidal geometry for complex.

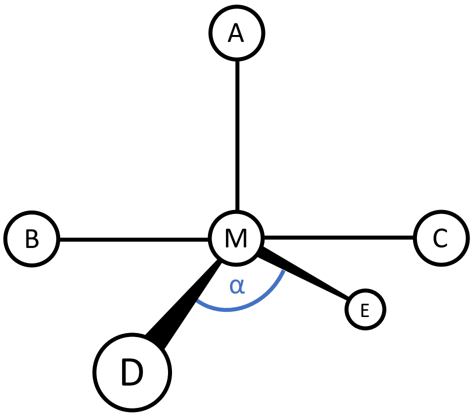

Supplement: Supplementary file 4 [file e-79-01109-sup5.pdf]
